# Fatty Acid Desaturation Links Germ Cell Loss to Longevity Through NHR-80/HNF4 in *C. elegans*

**DOI:** 10.1371/journal.pbio.1000599

**Published:** 2011-03-15

**Authors:** Jérôme Goudeau, Stéphanie Bellemin, Esther Toselli-Mollereau, Mehrnaz Shamalnasab, Yiqun Chen, Hugo Aguilaniu

**Affiliations:** Ecole normale supérieure de Lyon–CNRS–Université de Lyon Claude Bernard, Molecular Biology of the Cell Laboratory/UMR5239, Lyon, France; Brown University, United States of America

## Abstract

**Background:**

Preventing germline stem cell proliferation extends lifespan in nematodes and flies. So far, studies on germline-longevity signaling have focused on *daf-16/FOXO* and *daf-12/VDR*. Here, we report on NHR-80/HNF4, a nuclear receptor that specifically mediates longevity induced by depletion of the germ line through a mechanism that implicates fatty acid monodesaturation.

**Methods and Findings:**

*nhr-80/HNF4* is induced in animals lacking a germ line and is specifically required for their extended longevity. Overexpressing *nhr-80/HNF4* increases the lifespan of germline-less animals. This lifespan extension can occur in the absence of *daf-16/FOXO* but requires the presence of the nuclear receptor DAF-12/VDR. We show that the fatty acid desaturase, FAT-6/SCD1, is a key target of NHR-80/HNF4 and promotes germline-longevity by desaturating stearic acid to oleic acid (OA). We find that NHR-80/HNF4 and OA must work in concert to promote longevity.

**Conclusions:**

Taken together, our data indicate that the NHR-80 pathway participates in the mechanism of longevity extension through depletion of the germ line. We identify *fat-6* and OA as essential downstream elements although other targets must also be present. Thus, NHR-80 links fatty acid desaturation to lifespan extension through germline ablation in a *daf-16/FOXO* independent manner.

## Abstract 2

Lifespan extension induced by germline ablation in *C. elegans* is regulated by the nuclear hormone receptor NHR-80 in a process that requires the production of oleic acid by activation of the lipid desaturase FAT-6/SCD1.

## Author Summary

Reproduction and aging are two processes that seem to be closely intertwined. Experiments in *Caenorhabditis elegans* and *Drosophila* have shown that depletion of the germ line increases lifespan and that this process depends on insulin and lipophilic-hormone signaling. Recently, it was demonstrated that when germline stem cells (GSCs) cease to proliferate, fat metabolism is altered and this affects longevity. In this study, we have identified a nuclear hormone receptor, NHR-80, that mediates longevity through depletion of the germ line by promoting fatty acid desaturation. The *nhr-80* gene is up-regulated at the mRNA and protein levels in germline-less animals, leading to the transcription of the gene, *fat-6*, and the production of oleic acid (OA). Our experiments also show that the NHR-80/FAT-6/OA pathway does not require the presence of DAF-16 but instead, depends fully on the presence of DAF-12, a steroid receptor that affects lifespan. We provide evidence that other NHR-80 targets must be present concomitantly. Our results reinforce the notion that fat metabolism is profoundly altered in response to GSC proliferation, and the data contribute to a better understanding of the molecular relationship between reproduction, fat metabolism, and aging.

## Introduction

Removing the germ line of *Caenorhabditis elegans* extends its lifespan by approximately 60% [1]. Eliminating germ cells also increases the lifespan of *Drosophila*, suggesting that a conserved mechanism links the germ line to longevity [2]. In *C. elegans*, removal of the germ line can be achieved either by laser ablation of germline precursor cells at early developmental stages or through mutations that impair the proliferation of germline stem cells (GSCs) [3]. The *glp-1(e2141ts)* and *mes-1(bn7)* alleles deplete the germ line by either blocking proliferative signals for GSC or inhibiting cell division in the P lineage at early embryonic stages. As a result, animals carrying these alleles are long-lived [3]. Longevity is not merely caused by sterility because animals lacking both germ cells and the somatic gonad are sterile but not long lived. The germ line and the somatic gonad have been suggested to have opposite effects on longevity [1], but the molecular basis of germline-mediated longevity remains poorly understood.

Hsin and Kenyon showed that the presence of *daf-16/FOXO* or *daf-12/VDR* is required for extending the lifespan of animals whose germ line had been ablated [1]. DAF-16/FOXO is a forkhead transcription factor that translocates into intestinal nuclei and promotes transcription when GSCs stop proliferating [4]. *daf-16/FOXO* is a key downstream component of the insulin/IGF1 signaling (IIS) pathway that also regulates longevity [5],[6]. Although inhibiting GSC proliferation and down-regulating the activity of the IIS pathway both result in lifespan extension and translocation of DAF-16 into intestinal nuclei, several experiments show that these manipulations are not equivalent. First, GSC removal extends the lifespan of *daf-2* mutants that are already long-lived due to a constitutively down-regulated IIS pathway (hypomorphic allele of the sole insulin receptor) [1]. Nuclear translocation of DAF-16 requires the intestinal protein KRI-1 (an ankyrin-repeat protein) when it is provoked by a GSC proliferation arrest, but not by *daf-2* mutations [4]. Finally, the transcription elongation factor, TCER-1, promotes the transcriptional activity of *daf-16/FOXO* when GSCs stop dividing but not in long-lived IIS mutants [7]. Taken together, these data indicate that IIS and GSCs affect longevity though distinct mechanisms although they are both mediated by DAF-16.

The second pathway required for germline longevity is the DAF-12 lipophilic-hormone signaling pathway. In response to the loss of germ cells, the cytochrome P450, DAF-9 [8], and the Rieske protein, DAF-36 [9], use cholesterol to produce a steroid hormone (dafachronic acids) that activates the nuclear hormone receptor, DAF-12/VDR [10]. DAF-12/VDR is homologous to the vertebrate vitamin D receptor, and its presence in its activated form is required to extend lifespan though depletion of the germ line [1]. The interactions of KRI-1/DAF-16/TCER-1 and the DAF-9/DAF-36/DAF-12 pathways are still unclear.

Similar to KRI-1, DAF-9 and DAF-12 facilitate the nuclear translocation of DAF-16 triggered by germline removal [4], suggesting that the lipophilic-hormone signaling pathway may act upstream of DAF-16. However, recent work showed that DAF-12 and DAF-16 also function separately. First, germline-less animals in which DAF-16 is forced into intestinal nuclei still require *daf-12* to be long-lived [4]. Second DAF-12 and DAF-16 promote the expression of different gene sets [11]. The Kenyon lab showed that *sod-3* and *cdr-6* are DAF-16 and DAF-12 targets, respectively [11]. The *K04A8.5* lipase is induced in *glp-1(e2141ts)* mutant animals in a *daf-16* dependent manner but is not affected by *daf-12* [12]. Since the *K04A8.5* lipase is also required for lifespan extension, these results suggest that the KRI-1/DAF-16/K04A8.5 pathway can promote longevity independently of *daf-12* and that DAF-16 dependent transcription does not strictly require *daf-12*. Finally, it has also been shown that the DAF-12 lipophilic-hormone signaling pathway can mediate longevity in response to the somatic reproductive tissues, but this also requires the presence of DAF-16 [11]. Thus, it is still unclear whether DAF-12 can promote longevity in the absence of *daf-16*.

In the present study, we searched for new nuclear receptors that can mediate longevity of *C. elegans* through depletion of the germ line using an RNAi-based genetic screen. We report that *nhr-80/HNF4* is required for extending lifespan through germline removal, although it does not affect the lifespan of wild type animals. We show that NHR-80 is specific to this pathway since other longevity paradigms are not affected by a *loss-of-function* mutation of *nhr-80*. Moreover, the levels of NHR-80 increase in intestinal cells when germ cells are depleted. This increase is physiologically relevant because (1) overexpressing *nhr-80* further extends the lifespan of germline-less animals and (2) germline ablation leads to the *nhr-80* dependent up-regulation of the stearoyl-CoA desaturase (SCD), *fat-6*, that produces oleic acid (OA) from stearic acid and (3) increased fatty acid desaturation and OA production are necessary to extend the lifespan of germline-less animals. A link between fat metabolism and germline-mediated longevity has already been reported by the Ruvkun lab. In this recent report, the authors reported that the triacylglyceride (TAG) lipase (*K04A8.5*) is required for germline longevity [12]. Both *fat-6* and *K04A8.5* are induced in germline-less animals, and their inactivation by RNAi fully suppresses lifespan extension by depletion of the germ line. However, in contrast to the *K04A8.5* lipase that acts downstream of the KRI-1/DAF-16 pathway and independently of the DAF-9/DAF-36/DAF-12 lipophilic-hormone signaling pathway, we show that the NHR-80/FAT-6/OA pathway does not require the presence of *daf-16* but necessitates the presence of *daf-12*. Taken together, our data and that of Wang et al. are consistent with the conclusion that the lifespan benefits triggered by inhibiting GSC proliferation require a important modification of the metabolism of fat since the NHR-80/FAT-6/OA and the KRI-1/DAF-16/K04A8.4 pathways are activated independently to promote longevity though the activation of fat remodeling enzymes.

## Results

### *nhr-80* Is Required for Lifespan Extension through Depletion of the Germ Line

To identify new genes required for lifespan extension triggered by germline ablation, we screened for genes encoding for nuclear receptors (NHRs) using RNAi by feeding. We sought genes whose inactivation could suppress the lifespan of *glp-1(e2141ts)* mutant *C. elegans* without affecting the lifespan of wild type animals. Therefore, we compared the proportion of dead animals in *glp-1(e2141ts)* mutant and wild type animals after 20 d of RNAi treatment (starting at day 1 of adulthood). At 20 d, 50% to 60% of *glp-1(e2141ts)* mutants were alive, compared to less than 30% of wild type animals. In our screen, successful candidates lowered the survival of *glp-1(e2141ts)* mutants significantly but did not affect that of wild type animals at 20 d of adulthood. Of the 195 NHRs present in the Ahringer library (70% of all NHRs present in *C. elegans*), only one, *nhr-80*, in addition to our positive control (*daf-12*), reduced the lifespan of *glp-1(e2141ts)* mutants without affecting wild type lifespan. In wild type fertile animals, NHR-80 promotes fatty acid (FA) desaturation without affecting lifespan [13].

**Figure 1:**
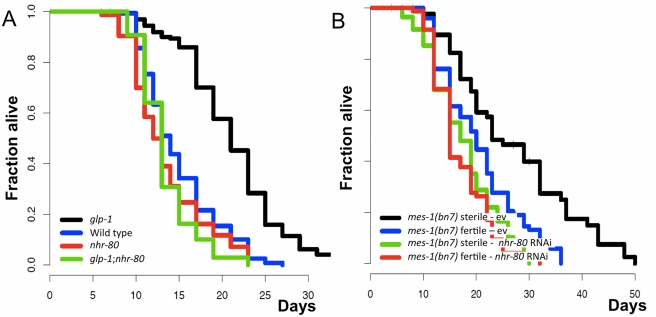
NHR-80 is required for lifespan extension caused by depletion of the germ line. (A) *nhr-80* is required for *glp-1(e2141ts)* mediated longevity (*p*<0.0001; 45% reduction in mean lifespan) but does not affect the lifespan of animals with a germ line (*p* = 0.55; mean lifespan of 15 d for wild type and *nhr-80(tm1011)*). All lifespan analyses were conducted under similar conditions, with a transient temperature shift to 25°C and without FU. (B) Lifespan of both sterile and fertile *mes-1(bn7)* mutants either subjected to *nhr-80* RNAi or not. Mean lifespan of the sterile *mes-1(bn7)* mutant is 21 d and 14 d when submitted to the empty vector (ev) and *nhr-80* RNAi, respectively (*p*<0.0001). Lifespan analyses were performed independently at least twice. The *p* values were calculated using the log rank (Mantel-Cox) analyses.

*C. elegans* carrying the *nhr-80(tm1011)* allele behave similarly to wild type animals subjected to *nhr-80* RNAi, suggesting that it is a *loss-of-function* mutation [13]. Although the *nhr-80(tm1011)* allele does not affect the lifespan of wild type animals (Figure 1A, Table S1), it fully suppresses that of *glp-1(e2141ts)* mutants (Figure 1A, Table S1), without restoring germline development (unpublished data). To ensure that down-regulation of *nhr-80* did not merely suppress the *glp-1(e2141ts)* allele, we examined whether *nhr-80* RNAi could also suppress another surrogate for germline-mediated longevity, *mes-1(bn7)* mutants. The lifespan extension observed in sterile *mes-1(bn7)* mutants is suppressed by *nhr-80* RNAi (Figure 1B, Table S1). This suggests that NHR-80 is required for extending longevity through depletion of the germ line.

### *nhr-80* Specifically Promotes Germline-Mediated Longevity

**Figure 2:**
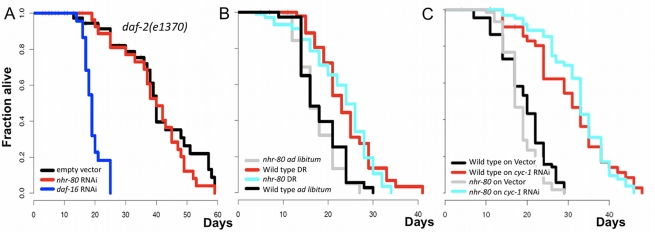
NHR-80 specifically promotes germline-mediated longevity. (A) *nhr-80* RNAi does not affect the lifespan of *daf-2(e1370)* mutants (*p* = 0.55), and (B, C) *nhr-80(tm1011)* mutants responded normally to both dietary restriction (*p*<0.0001, 7 d extension in mean lifespan, for *nhr-80(tm1011)* and wild type) and *cyc-1* RNAi treatment (*p*<0.0001, 15 and 14 d extension in mean lifespan, for *nhr-80(tm1011)* and wild type, respectively). Thus, *nhr-80* specifically mediates *glp-1(e2141ts)* longevity. Lifespan analyses were performed independently at least twice. The *p* values were calculated using the log rank (Mantel-Cox) analyses.

To determine whether *nhr-80* is specific to germline-mediated longevity, we knocked out *nhr-80* in several other longevity paradigms [5],[14],[15]. For example, *daf-2(e1370)* mutant animals are long-lived due to a *reduction-of-function* mutation in the insulin receptor and are not affected by *nhr-80* RNAi treatment (Figure 2A, Table S1). Similarly, dietary restriction (DR) and *cyc-1* RNAi, which reduces mitochondrial function [14], extend longevity to a comparable extent in *nhr-80(tm1011)* mutants and in wild type animals, suggesting that these pathways are not affected by NHR-80 (Figure 2C and D, Table S1). We conclude that NHR-80 specifically promotes germline longevity.

### The *nhr-80* mRNA and Protein Levels Increase in *glp-1(e2141ts)* Mutants

**Figure 3:**
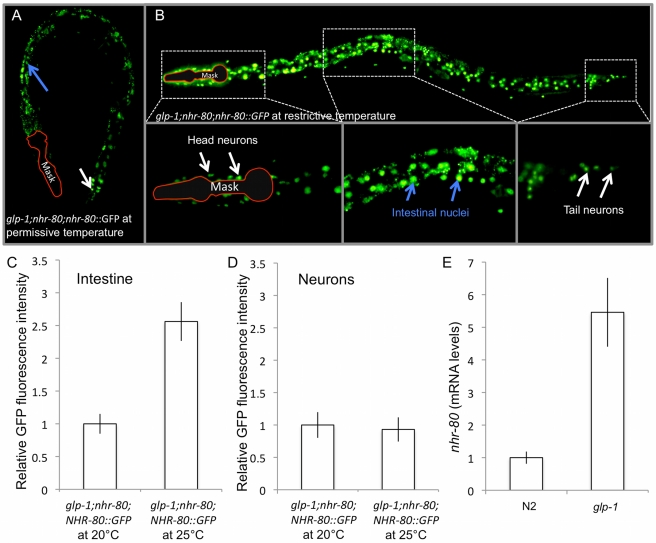
In response to germline depletion, *nhr-80* mRNA levels increase and NHR-80::GFP is induced in intestinal nuclei. (A and B) Using *glp-1(e2141ts)*;*lynEx[(nhr-80p::nhr-80::gfp) myo-2p::DsRed]* mutant animals, we observed that NHR-80 is expressed in the nuclei of head and tail neurons (white arrows) and of the intestine (blue arrows). Because of the high intensity of the *myo-2p::DsRed* marker, the pharynx was artificially masked, outlined in red. NHR-80::GFP is nuclear at both permissive and restrictive temperature. (C) The intensity of the signal is increased almost 1.6-fold at restrictive temperature in intestinal nuclei (Wilcoxon rank-sum test *p* value <0.001). (D) In contrast, when similar measurements were made in neuronal nuclei, no significant difference was detected (Wilcoxon rank-sum test *p* value is non-significant). (E) *nhr-80* mRNA levels are increased in *glp-1(e2141ts)* mutants as measured by qRT-PCR (5.6-fold increase; Wilcoxon rank-sum test *p* value <0.05 when compared with N2).

We analyzed the localization of NHR-80, using a functional protein fused to a GFP tag (see Materials and Methods and Figure 3B). In contrast to previous reports [16], we found that NHR-80 is localized in the nucleus and that it is expressed in the intestine and in neurons (some head and tail neurons, as well as the ventral cord; Figure 3A and B). This discrepancy is likely due to the fact that we fused a GFP tag to the full-length NHR-80 sequence driven by its own promoter, while Miyabayashi et al. fused the tag to the *nhr-80* promoter and may have missed the nuclear import signal [16]. We found that NHR-80 nuclear localization is constitutive and independent of the presence of the germ line (i.e., *glp-1(e2141ts);nhr-80(tm1011);NHR-80::GFP* mutant animals at restrictive versus permissive temperature) (Figure 3A and B). However, the intensity of the NHR-80::GFP in intestinal cells is increased by 60% when *glp-1(e2141ts);nhr-80(tm1011)* mutant animals are shifted to the restrictive temperature at the L1 stage while no changes are noted in neuronal cells (Figure 3C and D). When *glp-1(e2141ts)* mutant animals were shifted to restrictive temperature at later stages (L4 and day 1 of adulthood), the induction of intestinal NHR-80 still occurs, but less dramatically (L4 and day 1 of adulthood; Figure S1). Taken together with previous data showing that late shifts extend lifespan to a lesser extent [3], our data suggest that NHR-80 induction correlates with the extent to which lifespan is extended. To confirm this increase, we measured the overall expression level of *nhr-80* by qRT-PCR and found that it is induced 5.6-fold in *glp-1(e2141ts)* mutant animals (Figure 3E). Thus, in *glp-1(e2141ts)* mutant animals, overall *nhr-80* mRNA levels and the intensity of NHR-80::GFP in the intestine are increased. This strongly suggests that NHR-80 promotes longevity in the intestine. Supporting this notion, we found that *nhr-80* RNAi also suppresses the lifespan of *glp-1(e2141ts)* mutant animals, although neurons are refractory to RNAi (Figure S2, Table S1).

### Overexpressing *nhr-80* Increases the Lifespan of *glp-1(e2141ts)* Mutant Animals But Not That of Wild Type

**Figure 4:**
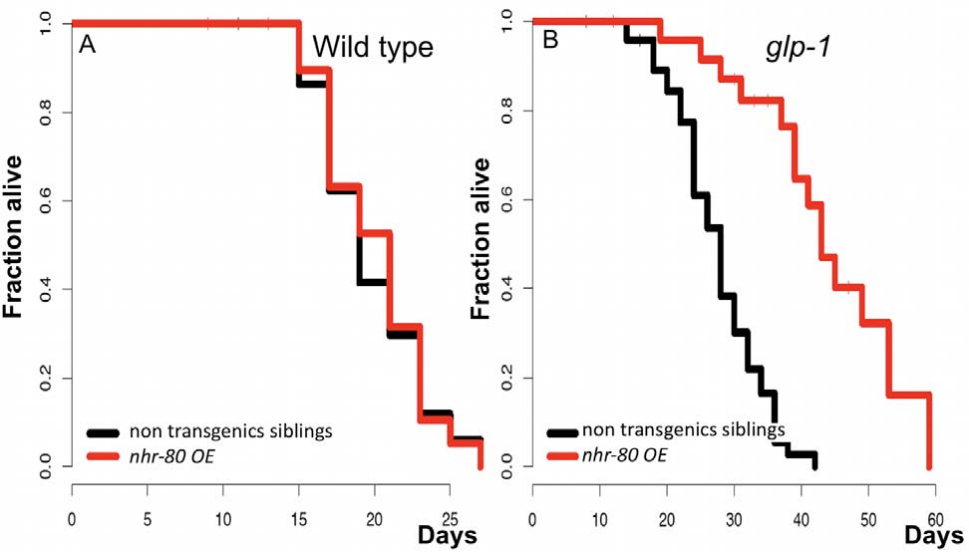
Overexpressing NHR-80 extends the lifespan of *glp-1(e2141ts)* mutants, but not of wild type animals. (A) In wild type animals, *lynEx[(nhr-80p::nhr-80::gfp) myo-2p::DsRed]* has no significant effect on longevity (mean lifespan of 16.5 and 17.5 for N2 non-transgenics siblings and overexpressing *nhr-80* (*nhr-80* OE*)*, respectively; *p* = 0.85). This suggests that overproducing NHR-80 is not sufficient to induce lifespan extension. (B) *nhr-80* overexpression (*lynEx[(nhr-80p::nhr-80::gfp);myo-2p::DsRed]*) increases the lifespan of *glp-1(e2141ts)* mutants (*p*<0.0001, 82% extension in mean lifespan). Lifespan analyses were performed independently at least twice. The *p* values were calculated using the log rank (Mantel-Cox) analyses.

Because *nhr-80* is a positive longevity regulator that is induced in *glp-1(e2141ts)* mutant animals, we examined whether overexpressing *nhr-80* could recapitulate germline-mediated longevity in a wild type context. Surprisingly, the *nhr-80* transgene, which fully restores the longevity of *glp-1(e2141ts);nhr-80(tm1011)* mutant animals (Figure S3, Table S1), fails to extend the lifespan of wild type animals (Figure 4A, Table S1) but increases the mean lifespan of *glp-1(e2141ts)* mutant animals by 80% (Figure 4B, Table S1). It is remarkable that a *loss-of-function* mutation and an overexpression of *nhr-80* have opposite effects on lifespan in the absence of proliferating GSCs only (Figures 1A and 4B, Table S1). The mechanism through which this is achieved remains to be determined. Our data do not allow discrimination between activation by binding of NHR-80 to a ligand, post-translational modifications, or interaction with a partner. To gain further insights into NHR-80 functions, we examined the interaction of *nhr-80* with other longevity determinants of germline-less animals.

### *nhr-80* Does Not Require *daf-16* to Promote Longevity

**Figure 5:**
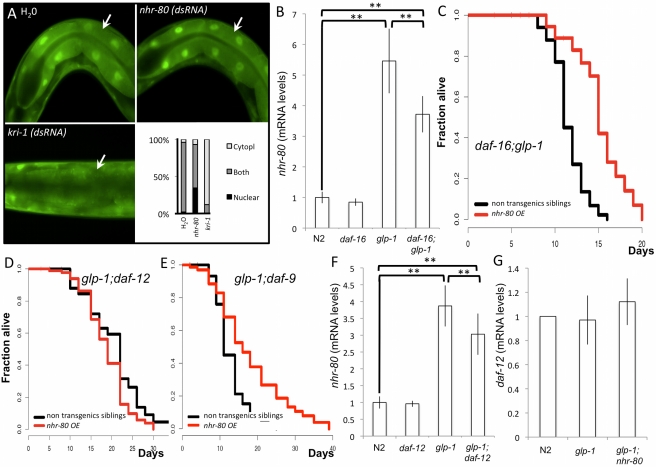
NHR-80 can function in the absence of *daf-16* but requires *daf-12*. (A) DAF-16 localization is not affected by *nhr-80*. Using the CF1935 strain (*daf-16(mu86)I;glp-1(e2141ts)III; muIs109)*, we assessed the localization of DAF-16::GFP by fluorescent microscopy. Fertile *daf-16(mu86);glp-1(e2141ts);muIs109 [Pdaf-16::gfp::daf-16]* mutant animals were injected with water (control), *kri-1*, or *nhr-80* dsRNA (1 mg/ml) as previously described [4]. Progeny of injected animals were assayed for DAF-16 localization in intestinal cells, indicated by a white arrow, using a fluorescent microscope (Axioplan, Zeiss). DAF-16::GFP is strongly nuclear in the progeny of water injected (upper left panel) or *nhr-80* dsRNA injected animals (upper right panel), while it remains cytoplasmic when *kri-1* dsRNA was injected (lower left panel). To ensure that injection of *nhr-80* ds RNA was efficient, lifespan of the progeny was monitored and was shortened as expected (unpublished data). Images are magnified 630-fold. The lower right panel shows a quantification of these results. Percent of animals showing either nuclear, cytoplasmic localization, or both is depicted on the graph (50, 29, and 25 animals were analyzed for water control, *nhr-80* (dsRNA), and *kri-1* (dsRNA), respectively). (B) *nhr-80* mRNA levels are increased in *glp-1(e2141ts)* and in *daf-16(mu86);glp-1(e2141ts)* mutants as measured by qRT-PCR (5.6- and 3.7-fold increase; Wilcoxon rank-sum test *p* value <0.05 for both strains when compared with N2). When *daf-16(mu86);glp-1(e2141ts)* mutants are compared to *glp-1(e2141ts)* mutants, there is a 1.5-fold decrease (Wilcoxon rank-sum test *p* value <0.05). Error bars are standard deviation (*p<0.1, **p<0.05, ***p<0.01, Wilcoxon rank-sum test). This suggests that *nhr-80* mRNA levels respond to *glp-1(e2141ts)* mutants and that part of this response is *daf-16* independent. A control experiment shows that *nhr-80* is not under the control of *daf-16* in the wild type background (Wilcoxon rank-sum test *p* value is non-significant). (C) *nhr-80* overexpression (*lynEx[(nhr-80p::nhr-80::gfp);myo-2p::DsRed]*) increases the lifespan of *daf-16(mu86)*;*glp-1(e2141ts)* mutant animals (*p*<0.0001, 38% extension in mean lifespan) but (D) fails to increase the lifespan of *glp-1(e2141ts);daf-12(rh61rh411)* double mutants (MLS of 18 and 19.5 d for *glp-1(e2141ts);daf-12(rh61rh411)* mutants carrying the transgene or not; *p* = 0.17). (E) Finally, *nhr-80* overexpression increases the lifespan of *glp-1(e2141ts);daf-9(rh50)* double mutants (MLS of 15 and 10 d for *glp-1(e2141ts);daf-9(rh50)* mutants carrying the transgene or not, respectively; *p*<0.0001, 50% extension in mean lifespan). Thus, while NHR-80 can partially bypass the need for DAF-16, its longevity function requires the presence of DAF-12, but not of DAF-9. Lifespan analyses were performed independently at least twice. The *p* values were calculated using the log rank (Mantel-Cox) analyses. (F) *nhr-80* mRNA levels are up-regulated in *glp-1(e2141ts)* and in *glp-1(e2141ts);daf-12(rh61rh411)* mutant animals (3.87- and 3.01-fold induction, respectively, compared to N2 treated similarly; Wilcoxon rank-sum test *p* value <0.05 for both; error bars are standard deviation). When compared to *glp-1(e2141ts)* mutants, *nhr-80* mRNA levels are decreased 1.2-fold in *glp-1(e2141ts);daf-12(rh61rh411)* (Wilcoxon rank-sum test *p* value <0.05; error bars are standard deviation). *nhr-80* induction does not strictly require DAF-12 but is modulated by DAF-12. (G) *daf-12* mRNA levels are unaffected in *glp-1(e2141ts)* and in *glp-1(e2141ts);nhr-80(tm1011)* mutant animals (1.07- and 1.10-fold induction, respectively, compared to N2 treated similarly; the Wilcoxon rank-sum test *p* value is non-significant; error bars are standard deviation).

To examine mechanisms through which NHR-80 may promote the longevity of germline-depleted animals, we first tested whether *nhr-80* longevity function depends on DAF-16. To address this question, we assessed whether NHR-80 may promote DAF-16 nuclear localization, similar to KRI-1 (orthologous to the human disease gene KRIT1; [4]). We found that down-regulating *nhr-80* by injection of double-stranded RNA does not affect the localization of DAF-16 (Figure 5A). Next, we tested whether *daf-16* was required for the increase of *nhr-80* mRNA levels triggered by the depletion of the germ line or the longevity extension provoked by *nhr-80* overexpression. We found that, in *daf-16(mu86);glp-1(e2141ts)* mutant animals, the *nhr-80* mRNA levels are increased relative to wild type (Figure 5B; 3.7-fold; *p* = 0.002) and that overexpressing *nhr-80* increases lifespan by 40% (Figure 5C, Table S1), while *nhr-80* RNAi decreases lifespan by 58% (Figure S4, Table S1). However, the transcriptional induction of *nhr-80* and the lifespan extension triggered by *nhr-80* overexpression are decreased in the absence of *daf-16* relative to *glp-1(e2141ts)* mutants, suggesting that DAF-16 can modulate the transcriptional induction of *nhr-80*. Thus, DAF-16 is not strictly required for *nhr-80* function, but DAF-16 can modulate *nhr-80* mRNA levels.

### NHR-80 Requires the Presence of DAF-12 to Promote Longevity

We observed that *nhr-80* overexpression fails to extend the lifespan of *glp-1(e2141ts);daf-12(rh61rh411)* mutants (Figure 5D, Table S1) but extends the lifespan of *glp-1(e2141ts);daf-9(rh50)* mutants (Figure 5E, Table S1). Thus, DAF-12 is required for NHR-80 mediated longevity, but not DAF-9. This suggests that NHR-80 functions independently of the DAF-9 derived ligand, Δ7-dafachronic acid [10]. To confirm this idea, we tested whether *nhr-80* overexpression could also extend lifespan of *glp-1(e2141ts);daf-9(rh50)* mutants in the presence of Δ7-dafachronic acid. We found that this was the case: in the presence of Δ7-dafachronic acid, *nhr-80* overexpression extends the lifespan of *glp-1(e2141ts);daf-9(rh50)* double mutants to the same extent (Figure S5, Table S1). These data confirm that NHR-80 genetically interacts with DAF-12 in a *daf-9* and Δ7-dafachronic acid independent manner.

To further explore how *daf-12* and *nhr-80* may interact, we measured the *nhr-80* mRNA levels in *glp-1(e2141ts);daf-12(rh61rh411)* mutant animals and found that *nhr-80* mRNA levels are induced in these animals relative to wild type (Figure 5F), suggesting that DAF-12 is not strictly required for the *nhr-80* transcriptional induction. However, similar to DAF-16, DAF-12 can modulate *nhr-80* mRNA levels (Figure 5F). This may be explained by the presence of two distant DAF-12 binding sites on the NHR-80 promoter (Figure S6). We also measured the *daf-12* mRNA levels to test whether DAF-12 could be a target of NHR-80 and found that they are not affected in a *glp-1(e2141ts);nhr-80(tm1011)* context (Figure 5G). Taken together, our data indicate that *nhr-80* and *daf-12* are not exclusive transcriptional targets of one another. The simplest way to explain this interaction is that DAF-12 and NHR-80 function in concert to promote longevity. The finding that *nhr-80* is functional in *glp-1(e2141ts);daf-9(rh50)* mutants shows that DAF-12 can promote longevity independently of *daf-9* in a germline-less animals.

### *fat-6* Is Induced in *glp-1(e2141ts)* Mutant Animals in a NHR-80 Dependent Manner

**Figure 6:**
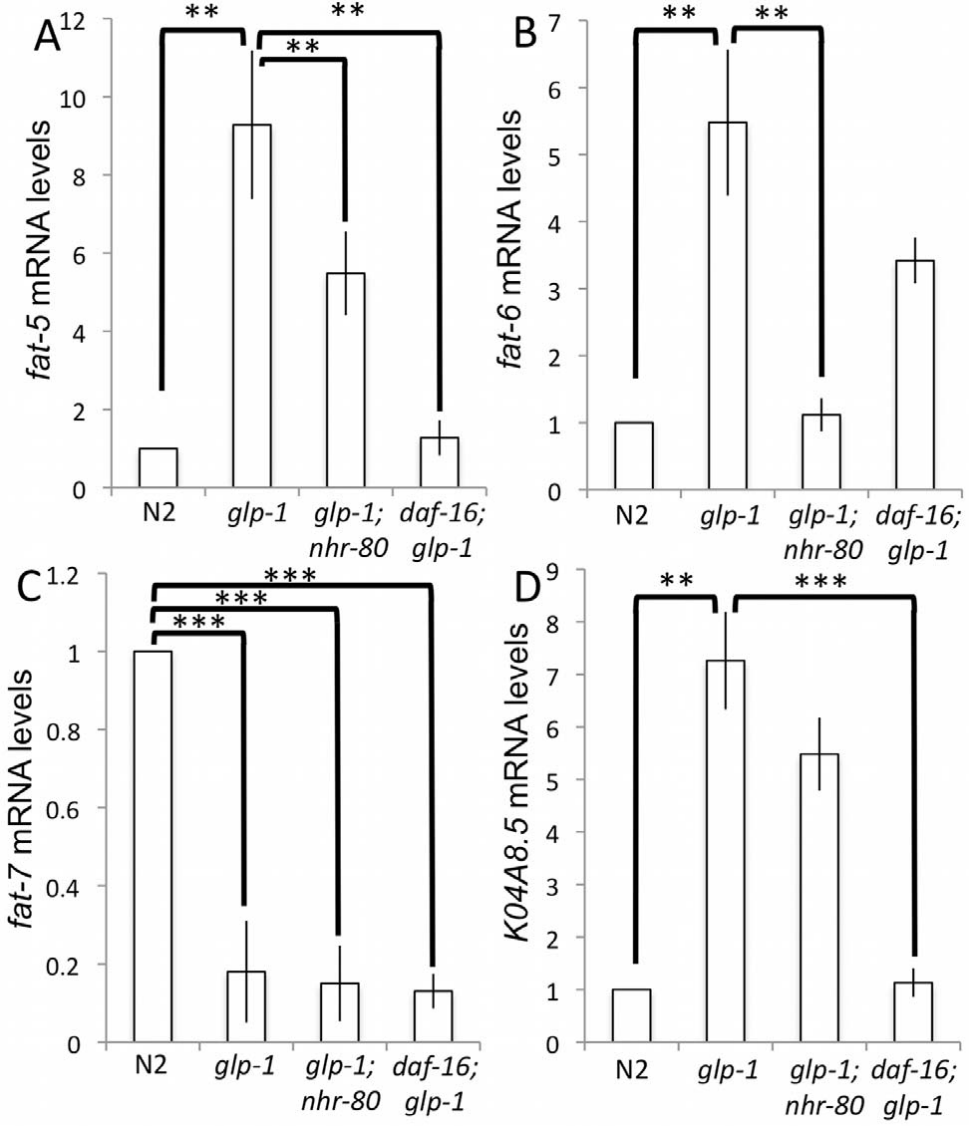
*fat-6* mRNA levels are induced through NHR-80 in *glp-1(e2141ts)* animals. (A) *fat-5* mRNA levels in wild type, *glp-1(e2141ts)*, *glp-1(e2141ts);nhr-80(tm1011)*, and *daf-16(mu86);glp-1(e2141ts)* mutants. (B, C, and D) Similar analyses were performed for *fat-6*, *fat-7*, and *K04A8.5*. *fat-5*, *fat-6*, and *K04A8.5* mRNA levels are up-regulated in *glp-1(e2141ts)* mutants compared to wild type (9.28-, 5.48-, and 7.25-fold, respectively; Wilcoxon rank-sum test *p* value <0.05 for all). In contrast, *fat-7* mRNA levels are decreased in *glp-1(e2141ts)* mutants (6.6-fold Wilcoxon rank-sum test *p* value <0.001). *fat-5* mRNA levels are reduced 7.28-fold in *daf-16(mu86);glp-1(e2141ts)* mutants and 1.7-fold in *glp-1(e2141ts);nhr-80(tm1011)* mutants compared to *glp-1(e2141ts)* mutants (Wilcoxon rank-sum test *p* value <0.05 for both). When compared to wild type levels, *fat-5* is induced 1.29-fold in *daf-16(mu86);glp-1(e2141ts)* mutants and 5.48-fold in *glp-1(e2141ts);nhr-80(tm1011)* double mutants (Wilcoxon rank-sum test *p* value <0.05 for both; error bars are standard deviation; *p<0.1, **p<0.05, ***p<0.01, Wilcoxon rank-sum test). In contrast, *fat-6* mRNA levels are reduced 1.6-fold in a *daf-16(mu86);glp-1(e2141ts)* mutant and 4.9-fold in *glp-1(e2141ts);nhr-80(tm1011)* mutants compared to *glp-1(e2141ts)* mutants (Wilcoxon rank-sum test *p* value <0.05 and non-significant, respectively). When compared to wild type levels, *fat-6* is induced 2.9-fold in *daf-16(mu86);glp-1(e2141ts)* double mutants and 0.96-fold in *glp-1(e2141ts);nhr-80(tm1011)* double mutants (Wilcoxon rank-sum test *p* value <0.05 and non-significant, respectively; error bars are standard deviation; *p<0.1, **p<0.05, ***p<0.01, Wilcoxon rank-sum test). Relative to *glp-1(e2141ts)* mutants, *K04A8.5* is decreased 6.4-fold in *daf-16(mu86);glp-1(e2141ts)* mutants (*p*<0.01) and 1.4-fold in *glp-1(e2141ts);nhr-80(tm1011)* double mutants (Wilcoxon rank-sum test *p* value is non-significant). Finally, we found that *fat-7* mRNA levels are unchanged in *daf-16(mu86);glp-1(e2141ts)* and in *glp-1(e2141ts);nhr-80(tm1011)* mutants relative to *glp-1(e2141ts)* mutants (Wilcoxon rank-sum test *p* value is non-significant). Thus, the induction of *fat-5* and *K04A8.5* is DAF-16 dependant while that of *fat-6* depends on NHR-80 only.

Next, we examined the role of NHR-80 targets. Known transcriptional targets of NHR-80 in a wild type context include three Δ9-desaturases involved in lipid metabolism: *fat-5*, *fat-6*, and *fat-7* [13]. *fat-5* is a *p*almitoyl-*C*oA- Δ9-*d*esaturase (PCD) that converts palmitic acid to palmitoleic acid while *fat-6* and *fat-7* are *s*tearoyl-*C*oA- Δ9-*d*esaturases (SCD) that convert stearic acid to OA [13]. We measured mRNA levels of these three targets and of the lipase K04A8.5, another enzyme involved in lipid metabolism and germline-mediated longevity [12]. We found that, in *glp-1(e2141ts)* mutants, *fat-5*, *fat-6*, and *K04A8.5* are strongly induced, while *fat-7* is repressed (Figure 6A, 6B, 6C, and 6D). In *glp-1(e2141ts);nhr-80(tm1011)* mutants, the induction of *fat-6* is abolished (*p*≤0.001), *fat-5* is suppressed to a lesser extent (*p* = 0.002) and *K04A8.5* and *fat-7* are unaffected (Figure 6A, 6B, 6C, and 6D). Because lipid desaturases have been shown to be transcriptional targets of DAF-16 in *daf-2* mutants [17], we also measured the mRNA levels of *fat-5*, *fat-6*, *fat-7*, and *K04A8.5* in *daf-16(mu86);glp-1(e2141ts)* mutant animals. In this background, the induction of *fat-5* and *K04A8.5* no longer occurs (*p* = 0.005; Figure 6A and 6D), but that of *fat-6* or *fat-7* was not affected (*p* = 0.3; Figure 6B and 6C). Thus, our data indicate that *fat-6* transcriptional up-regulation depends on NHR-80, while that of *fat-5* depends on both NHR-80 and DAF-16. As previously reported, *K04A8.*5 is a DAF-16 target [12], but not of NHR-80 (Figure 6B). This suggests that, in the *glp-1(e2141ts)* context, *fat-6* is a NHR-80 target. To verify whether the *nhr-80* dependent up-regulation of *fat-6* results in an increased production of OA, we measured the OA concentration in fertile and germline-less animals. We found that the levels of OA as well as the stearic/oleic acid ratio are specifically increased in germline-less animals (Figure S7). This confirms the up-regulation of the FAT-6/OA pathway in *glp-1(e2141ts)* mutants.

### Stearoyl-CoA-Δ9-Desaturase Activity Is Required for *glp-1(e2141ts)* Longevity

**Figure 7:**
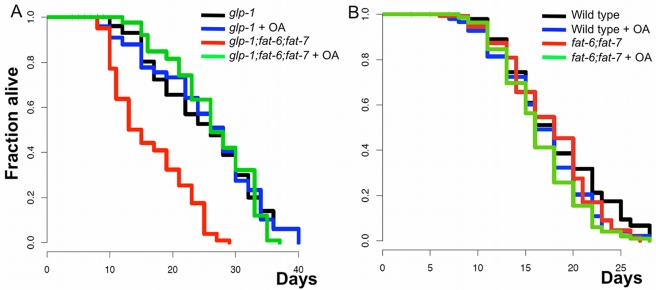
Stearoyl Co-A Δ9 Desaturase Activity Is Required for Germline-Mediated Longevity. (A) Eliminating the SCD activity by deleting both *fat-6* and *fat-7* in *glp-1(e2141ts)* mutants decreases its lifespan significantly (*p*<0.001, 46% reduction in mean lifespan). Addition of oleic acid (OA) during the whole course of the experiment increases the lifespan of *glp-1(e2141ts);fat-6(tm331);fat-7(wa36)* mutant animals (*p*<0.001, 108% increase in mean lifespan compared to untreated *glp-1(e2141ts);fat-6(tm331);fat-7(wa36)* mutants while it did not affect the lifespan of *glp-1(e2141ts)* mutants (*p* = 0.96, MLS of 25 d with and without OA)). To bypass potential developmental arrests or delays of *glp-1(e2141ts);fat-6(tm331); fat-7(wa36)* mutants, OA was provided to all strains until day 1 of adulthood in this experiment. (B) *fat-6(tm331);fat-7(wa36*) mutants have a normal lifespan and are not affected by the addition of oleic acid (MLS are 17 d for N2, *fat-6(tm331); fat-7(wa36*) with or without OA and 16.5 for N2 with OA). To bypass potential developmental arrest or delays for *fat-6(tm331); fat-7(wa36)*, OA was provided to all strains until day 1 of adulthood in this experiment. Lifespan analyses were performed independently at least twice. The *p* values were calculated using the log rank (Mantel-Cox) analyses.

To investigate the relevance of each Δ9-desaturase to *glp-1(e2141ts)* longevity, we deactivated all of the *fat* genes individually in a *glp-1(e2141ts)* background. We found that deletion of the fat genes independently in *glp-1(e2141ts)* mutant animals does not affect longevity (Figure S8, Table S1). However, using qRT-PCR, we found that, similar to other reports in wild type animals [18], compensatory mechanisms occur between *fat-6* and *fat-7* in germline-depleted animals and *fat-7* mRNA levels are strongly up-regulated in *glp-1(e2141ts);fat-6(tm331)* mutant animals (*p*≤0.001; Figure S9). To bypass this compensatory mechanism, we generated the triple mutants *glp-1(e2141ts);fat-6(tm331);fat-7(wa36)* (SCD activity is fully abolished), *glp-1(e2141ts);fat-6(tm331);fat-5(tm420)*, and *glp-1(e2141ts);fat-7(wa36)*;*fat-5(tm420)*. Concomitant deletion of *fat-5* with one of the SCDs (*fat-6* or *fat-7*) either slightly increases or decreases the lifespan of *glp-1(e2141ts)* mutant animals, while it does not affect the lifespan of wild type animals (27 and 20 d for *glp-1(e2141ts);fat-7(wa36)*;*fat-5(tm420)* and *glp-1(e2141ts);fat-6(tm331);fat-5(tm420)* mutants, respectively, versus 26 d for *glp-1(e2141ts)* mutants; Figure S10, Table S1). In contrast, deleting two SCDs (*fat-6* and *fat-7*) sharply decreases the longevity of *glp-1(e2141ts)* mutant animals without affecting that of wild type animals (MLS = 14 d for *glp-1(e2141ts);fat-6(tm331);fat-7(wa36)* mutants; Figure 7A and B, Table S1). Importantly, the addition of OA, the product of the reaction catalyzed by FAT-6/FAT-7, during adulthood in *glp-1(e2141ts);fat-6(tm331);fat-7(wa36)* mutants restores the lifespan of these animals (MLS = 26 d for supplemented mutants and 25 d for *glp-1(e2141ts)* mutants), while it does not affect the lifespan of either *fat-6(tm331);fat-7(wa36)* mutant or wild type animals (Figure 7A and B, Table S1). This suggests a key role for OA or one of its metabolites in linking germ cell loss to longevity. Next, we showed that blocking further processing of OA by desaturation to poly-unsaturated fatty acids using *fat-2* RNAi does not affect the lifespan of *glp-1(e2141ts)* mutants (Figure S11A, Table S1). Finally, we note that OA fails to extend the lifespan of *glp-1(e2141ts)* mutant animals. This is surprising because overexpressing *nhr-80* significantly increases the lifespan of these animals (Figure 7A, Table S1). Our data therefore indicate that NHR-80 promotes longevity through both the FAT-6/OA branch and other critical targets. We conclude that SCD activity and OA itself is required, but not sufficient, for germline-mediated longevity.

### Oleic Acid Does Not Extend the Lifespan of Either *daf-16(mu86);glp-1(e2141ts)* or *glp-1(e2141ts);daf-12(rh61rh411)* Mutants

The finding that OA is produced in response to the NHR-80/FAT-6 pathway provides an additional possibility to test the interaction of the FAT-6/OA branch with the other main germline longevity pathways: the KRI-1/DAF-16*/*K04A8.5 and the DAF-9/DAF-12 lipophilic-hormone pathways. Since our results demonstrated that KRI-1/DAF-16/K04A8.5 acts independently from the NHR-80/FAT-6 pathway, we verified whether OA could extend the lifespan of *daf-16(mu86);glp-1(e2141ts)* mutant animals and found that it fails to do so (Figure S12, Table S1). This confirms that addition of exogenous OA is not equivalent to *nhr-80* overexpression, which extends the lifespan of the *daf-16(mu86);glp-1(e2141ts)* double mutants (Figure 5C, Table S1) and indicates that FAT-6 is not the only NHR-80 target that promotes longevity. It is consistent with the idea that OA is already highly produced in *glp-1(e2141ts)* and *daf-16(mu86);glp-1(e2141ts)* mutants because the NHR-80/FAT-6/OA is already effective.

Next, we tested the effect of OA on the lifespan of *glp-1(e2141ts);daf-12(rh61rh411)* and *glp-1(e2141ts);daf-9(rh50)* mutants and found that it fails to extend lifespan in these two backgrounds (Figure S12, Table S1). Furthermore, we found that *fat-6* mRNA levels are still induced in *glp-1(e2141ts);daf-12(rh61rh411)* mutant relative to wild type animals (Figure S13). Taken together, our data exclude the possibility that the FAT-6/OA branch is a common target of DAF-12 and NHR-80. Thus, we conclude that DAF-12 does not interact with NHR-80 by co-promoting FAT-6.

### NHR-80 and Oleic Acid Promote Germline-Mediated Longevity in Concert

**Figure 8:**
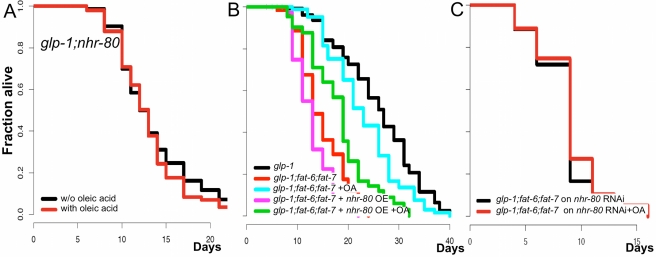
NHR-80 and oleic acid promote germline-longevity in concert. (A) Addition of OA fails to increase the lifespan of *glp-1(e2141ts);nhr-80(tm1011)* double mutants (*p* = 0.27, 14 d mean lifespan with or without OA). This demonstrates that NHR-80 is required for OA mediated lifespan extension. (B) *nhr-80* overexpression (*lynEx[(nhr-80p::nhr-80::gfp);myo-2p::DsRed]*) fails to increase the lifespan of *glp-1(e2141ts);fat-6(tm331);fat-7(wa36)* triple mutants (MLS of 12 d for *glp-1(e2141ts);fat-6(tm331);fat-7(wa36)* mutants carrying the transgenic or not; *p* = 0.07). Addition of exogenous OA increases the lifespan of *glp-1(e2141ts);fat-6(tm331);fat-7(wa36)* mutants carrying the *nhr-80* transgene, confirming that OA is required for the function of NHR-80 (MLS of 12 and 23 d for *glp-1(e2141ts);fat-6(tm331);fat-7(wa36)* mutants carrying the *nhr-80* transgene with or without OA, *p*<0.001). (C) OA no longer extends the lifespan of *glp-1(e2141ts);fat-6(tm331);fat-7(wa36)* triple mutants (MLS of 9 d for *glp-1(e2141ts);fat-6(tm331);fat-7(wa36)* mutants submitted to *nhr-80* RNAi with or without OA; *p* = 0.13). Lifespan analyses were performed independently at least twice. The *p* values were calculated using the log rank (Mantel-Cox) analyses.

**Figure 9:**
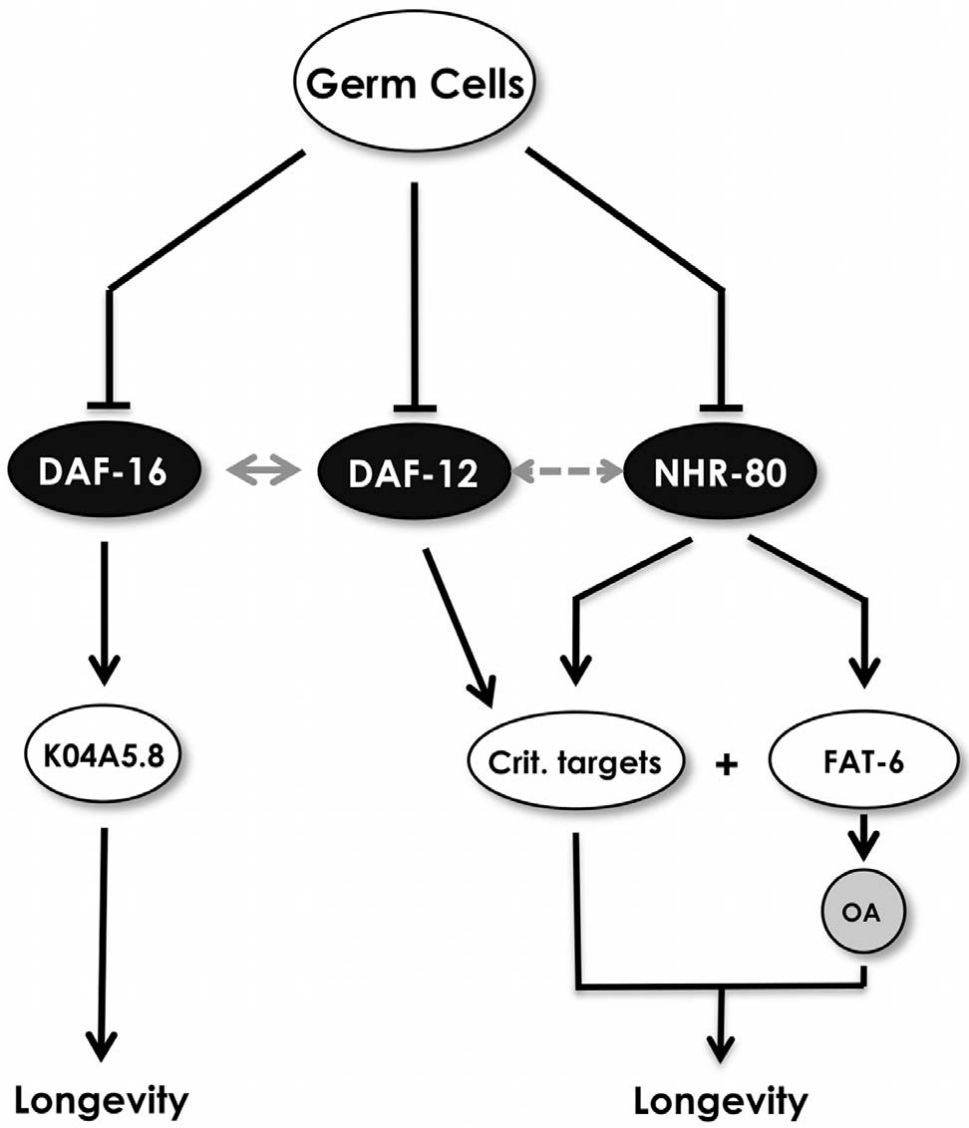
Model for NHR-80 function. Proliferating GSCs negatively regulate the translocation of DAF-16 within intestinal nuclei, the activation of DAF-12, and the expression of *nhr-80*. DAF-12 is central because it participates in the translocation of DAF-16 into intestinal nuclei (grey arrow) and is required for NHR-80′s longevity action. In response to depletion of the germ line, NHR-80 is up-regulated and becomes transcriptionally functional. *fat-6* is one of its targets and it encodes for a Stearoyl Co-A Δ9 Desaturase that produces OA. OA production is required, but not sufficient to promote longevity in the absence of proliferating GSCs. Thus, the FAT-6/OA acts in concert with other NHR-80 critical targets (Crit. targets). The DAF-16/K04A5.8 and the NHR-80 pathways can act independently, but DAF-12 is required for NHR-80 function. DAF-12 and NHR-80 do not interact at a transcriptional level and we propose that DAF-12 and NHR-80 targets interact to promote longevity (*fat-6* is not a DAF-12 target). The critical targets could be shared by DAF-12 and NHR-80 or distinct. Alternatively, DAF-12 may physically interact directly with NHR-80 (grey double head arrow).

Because our data suggest that OA and overexpressing *nhr-80* are distinct interventions, we next verified whether NHR-80 and OA must work in concert to mediate germline longevity. To address this, we first investigated whether OA could extend lifespan in the absence of *nhr-80*. Convincingly, the lifespan of *glp-1(e2141ts);nhr-80(tm1011)* mutant animals is not affected by OA (Figure 8A, Table S1). Conversely, we overexpressed *nhr-80* in animals that could no longer produce OA (*glp-1(e2141ts);fat-6(tm331);fat-7(wa36)*) and found that it fails to increase the lifespan of these animals (Figure 8B, Table S1). In this experiment, we noted that overexpressing *nhr-80* does not increase the lifespan of *glp-1(e2141ts);fat-6(tm331);fat-7(wa36*) mutants complemented with OA. This is surprising because *nhr-80* overexpression increases the lifespan of *glp-1(e2141)ts* mutants (Figure 4B, Table S1). The reason for this is unclear but may be explained by the fact that OA is not as efficient when it is externally provided since it is prone to oxidation. Finally, we showed that the effect of OA on the lifespan of *glp-1(e2141ts);fat-6(tm331);fat-7(wa36)* mutant animals is no longer significant when *nhr-80* is knocked down by RNAi (Figure 8C, Table S1). Taken together, our data suggest that OA and NHR-80 promote longevity in concert. This can be simply explained by the assumption that the OA producing pathway is not the only longevity-promoting branch downstream of NHR-80 (Figure 9).

## Discussion

In the present study, we show that, when germ cells are removed, *nhr-80* mRNA and protein levels increase. This promotes the mono-desaturation of stearic acid to OA by inducing the transcription of the stearoyl-CoA-desaturase, *fat-6/SCD1*. This cascade is physiologically relevant to longevity since both *nhr-80* and the SCD activity are required to augment the lifespan of germline-depleted animals. Furthermore, the lack of SCD activity can be bypassed by addition of exogenous OA in the medium, confirming the pivotal role of this metabolite.

Our data also indicate that the FAT-6/OA branch is required, but not sufficient, to promote longevity in response to depletion of the germ line downstream of NHR-80. This is evidenced by the fact that overexpressing *nhr-80* extends the lifespan of both *glp-1(e2141ts)* and *daf-16(mu86);glp-1(e2141ts)* mutants but OA does not, suggesting that these two interventions are not equivalent (Figures 4B, 5C, 7A, and S13A, Table S1). Supporting this view, we showed that OA and NHR-80 must act in concert to support the lifespan extension conferred by germ cell loss. Indeed, OA does not increase the lifespan of *glp-1(e2141ts);nhr-80(tm1011)* mutants and overexpressing *nhr-80* is inefficient when the SCD genes are deleted (OA producing genes). Moreover, while OA restores the lifespan of *glp-1(e2141ts);fat-6(tm331);fat-7(wa36)* mutant animals, it essentially fails to do so when *nhr-80* is deactivated by RNAi (Figure 8C, Table S1). Thus, our data support the notion that the OA production pathway is not the only longevity-promoting branch downstream of NHR-80. Finding other lifespan promoting NHR-80 targets will be an important goal in the future.

Our data are also compatible with the non-exclusive hypothesis that OA may act as a NHR-80 ligand. Although we do not provide biochemical evidence for this interaction in *C. elegans*, several articles have shown that long chain free fatty acids act as a ligand for the NHR-80 homolog in *Drosophila* and mammals, HNF4 [19]–[23]. It will be interesting to critically test this possibility in the future by either performing structural studies or transactivation assays to test the binding of OA to the NHR-80 ligand binding domain.

### The NHR-80/FAT-6/OA and the KRI-1/DAF-16/K04A8.5 Pathways Are Independent

Our results suggest that lipid metabolism and, in particular, fatty acid desaturation links signals from the germ line to longevity. This confirms previous findings suggesting a link between fat metabolism and longevity in germline-less animals through the K04A8.5 lipase [12]. However, results presented here argue that the NHR-80/FAT-6/OA and the KRI-1/DAF-16/K04A8.5 pathways can act independently. First, we observed that the increase in *nhr-80* mRNA levels observed in *glp-1(e2141ts)* mutant animals still occurs in the absence of *daf-16* (Figure 5B). Second, the translocation of DAF-16 into intestinal nuclei occurs in the absence of *nhr-80* (Figure 5A). Third, we show that NHR-80 and DAF-16 have distinct transcriptional targets, although some overlap between the two transcription factors exists. The transcription of *fat-6* is elevated in *glp-1(e2141ts)* mutant animals in a NHR-80 dependent manner (Figure 6B) and *K04A8.5* mRNA levels are increased in a DAF-16 dependent way (Figure 6D; [12]). *fat-5* is also increased, but its induction relies on both DAF-16 and NHR-80 (Figure 6A). Fourth, the overexpression of *nhr-80* increases the lifespan of *daf-16(mu86);glp-1(e2141ts)* mutant animals (Figure 5C, Table S1), demonstrating that NHR-80 signaling does not require the presence of *daf-16*. Finally, OA does not increase the lifespan of *daf-16(mu86);glp-1(e2141ts)* mutants, suggesting that, similar to *glp-1(e2141ts)* mutants, the SCD activity is already elevated in these animals (Figure S12A, Table S1).

Thus, our data confirm that germline ablation leads to an alteration of fat metabolism that is required for extending lifespan but challenge the view that this is centered on insulin signaling. Germ cell removal extends longevity in response to two independent fat modifying pathways: the NHR-80/FAT-6/OA and the KRI-1/DAF-16/K04A8.5 pathways.

### The NHR-80/FAT-6/OA Pathways Requires the Presence of DAF-12

DAF-12 is also required for longevity extension by depletion of the germ line [1], and it seems to act upstream of DAF-16 since its presence is partially required for DAF-16 translocation into intestinal nuclei in response to germline ablation [4]. However, recent observations suggest that DAF-12 can also act in parallel to DAF-16 [11],[12]. Our data show that the NHR-80/FAT-6/OA and DAF-12 act in concert, independently of DAF-16. Indeed, overexpressing *nhr-80* and providing exogenous OA do not affect the lifespan of *glp-1(e2141ts);daf-12(rh61rh411)* mutant animals (Figures 5D, S13B, Table S1), indicating that the NHR-80/FAT-6/OA pathway requires the presence of DAF-12. The expression levels of *daf-12* are not affected in *glp-1(e2141ts);nhr-80(tm1011)* mutants and the induction of *nhr-80* mRNA levels occurs in the absence of *daf-12*, but it is slightly decreased relative to *glp-1(e2141ts)* mutants, suggesting that, similar to DAF-16, DAF-12 modulates the NHR-80 response (Figure 5F). Our data therefore indicate that the DAF-12/NHR-80 interaction is not strictly transcriptional. Rather, we propose that DAF-12 and NHR-80′s targets act together to promote longevity or that NHR-80 and DAF-12 share some critical targets for longevity. Since *fat-6* mRNA levels are normally induced in *glp-1(e2141ts);daf-12(rh61rh411)* double mutants relative to wild type, we can exclude the possibility that the FAT-6/OA branch acts downstream of DAF-12. Thus, other targets must be involved. We propose a model where DAF-12 and NHR-80 targets cooperate to promote longevity in concert with the FAT-6/OA branch. However, it is also possible that DAF-12 interacts directly with NHR-80. Whether DAF-12 and NHR-80 only cooperate through their critical targets or whether they physically interact remains to be determined.

### The NHR-80/FAT-6/OA Pathway Does Not Require the Activation of Lipophilic-Hormone Signaling Pathway by Dafachronic Acid

We were surprised to find that overexpressing *nhr-80* extends the lifespan of *glp-1(e2141ts);daf-9(rh50)* double mutants in the presence or in the absence of Δ7 dafachronic acid, the DAF-12 ligand produced by DAF-9 ([10]; Figures 5E and S5, Table S1). This suggests that NHR-80 interacts with DAF-12 independently of DAF-9 and Δ7 dafachronic acid and explains why treating wild type animals overexpressing *nhr-80* with Δ7 dafachronic acid (DAF-12 ligand) fails to recapitulate germline longevity (unpublished data). We note that the Kenyon lab had already suggested that DAF-12 might function in a DAF-9 independent manner [4]. Indeed, mutations causing DAF-16 to be constitutively nuclear extends the lifespan of germline-less animals lacking *daf-9*, but not of animals lacking *daf-12* [4].

It is possible that DAF-12 can also be activated by other ligands or that it can interact with NHR-80 under its unliganded form. DAF-12 might also be activated by an unknown cofactor or by a heterodimeric partner nuclear receptor to interact with NHR-80. The finding that the overexpression of *nhr-80* fails to extend the lifespan of wild type animals (where DAF-12 is not activated; Figure 4A, Table S1) suggests indeed that other ligand(s) or co-activators may only be present in germline-less animals. However, we cannot exclude that other, yet unidentified, modulators preclude lifespan extension through *nhr-80* overexpression. In the future, it will be interesting to explore these non-exclusive possibilities.

### Fat Composition, Not Fat Content, Correlates with Germline Longevity

Similar to *daf-2* mutant animals, germline-depleted animals store more fat than wild type animals [24]. This is not a systematic trait of long-lived animals since diet-restricted animals store less fat. Thus, higher fat content is not a general cause for life extension. However, it is still not clear whether high fat content extends lifespan when the germ line is depleted. First, the high fat content phenotype [24] is hard to reconcile with the finding that the *K04A8.5* lipase is induced in these animals [12]. Although RNAi against the *K04A8.5* lipase increases Nile Red staining in *glp-1(e2141ts)* mutant animals, these data are difficult to interpret since it was shown that Nile Red does not stain fat reliably [24]. It is possible that the K04A8.5 lipase changes the composition of fat rather than altering overall fat content by degrading a subset of TAGs only. Second, the *nhr-80(tm1011)* allele does not affect the overall fat content of *glp-1(e2141ts)* mutants (unpublished observation). This indicates that fat content is not the cause for longevity extension in germline-depleted animals since *glp-1(e2141ts);nhr-80(tm1011)* mutants are short-lived. Our data suggest that fatty acid desaturation, and therefore, fat composition, is altered in germline-less animals. This modification of fat composition does not directly impact fat content but correlates with longevity. Indeed, our data clearly establish that OA production is not sufficient to extend lifespan, but it is strictly required. Further work should aim at understanding all aspects of fat metabolism that matter to germline-mediated longevity.

### A Conserved Mechanism?

Although *nhr-80* is one of 269 genes encoding nuclear hormone receptors in *C. elegans* that are homologous to the mammalian HNF4 gene [25], there are interesting clues that suggest that the mechanism we describe in this work may be conserved. First, we report that NHR-80 and OA must act in concert to promote longevity. Although we failed to provide clear evidence that OA activates NHR-80, structural and biochemical data showing a direct interaction between long chain fatty acids and HNF4 in *Drosophila* and mice [19]–[23] suggest that a similar regulation may also occur in *C. elegans* and that OA may be an NHR-80 ligand. Second, we show that *fat-6* is a target of NHR-80. Strikingly, SCD1, the mammalian homolog of FAT-6, is strongly up-regulated in response to ovariectomy in mice [26] and is a known target for several nuclear receptors in mammals. Taken together, these data suggest that our work may be relevant in mammalian systems. Further experiments are underway to firmly identify the NHR-80 analog.

## Materials and Methods

### Worm Maintenance and Strains

N2 Bristol was used as the wild-type strain. Nematodes were grown and maintained under standard conditions [27]. HGA8011, HGA8013, and BX156 were grown in the presence of Oleic acid until day 1 of adulthood to bypass any developmental delays. *C. elegans* strains (i.e., genotype, origin, strain name) are listed below:

*nhr-80(tm1011)III,* CGC*, BX165

*fat-5(tm420)V,* CGC, BX107

*fat-6(tm331)IV,* CGC, BX106

*fat-7(wa36)V*, CGC, BX153

*fat-6(tm331)IV;* fat-7(wa36)V, CGC, BX156

*fat-6(tm331)IV; fat-5(tm420)V, *CGC, BX160

*fat-7(wa36)V; fat-5(tm420)V, *CGC, BX110

*daf-12(m20)X,* CGC, DR20

*daf-16 (mu86)I,* CGC, AD105

*mes-1(bn7)X, *CGC, SS149

*glp-1(e2141ts)III*, Gift from Kenyon Lab, CF1903

*glp-1(e2141ts)III; daf-12(rh61rh411)X,* Gift from Kenyon Lab, CF1658

*glp-1(e2141ts)III; daf-9(rh50)X,* Gift from Kenyon Lab, CF1916

*daf-16(mu86)I; glp-1(e2141ts)III;* muIs109, Gift from Kenyon Lab, CF1935

*daf-16(mu86)I; glp-1(e2141ts)III, *Gift from Kenyon Lab, CF1880

*glp-1(e2141ts)III; nhr-80(tm1011)III,* Made in our Lab, HGA8000

N2; lynEx**, made in the laboratory, HGA8001

*glp-1(e2141ts)III;* lynEx, made in the laboratory, HGA8002

*glp-1(e2141ts)III; nhr-80(tm1011)III;* lynEx, made in the laboratory, HGA8003

*daf-16(mu86)I; glp-1(e2141ts)III;* lynEx, made in the laboratory, HGA8004

*glp-1(e2141ts)III;daf-12(rh61rh411)X;* lynEx, made in the laboratory, HGA8005

*glp-1(e2141ts)III; fat-5(tm420)V*, made in the laboratory, HGA8006

*glp-1(e2141ts)III; fat-6(tm331)IV*, made in the laboratory, HGA8007

*glp-1(e2141ts)III; fat-7(wa36)V,* made in the laboratory, HGA8008

*glp-1(e2141ts)III; fat-7(wa36)V;* fat-5(tm420)V, made in the laboratory, HGA8009

*glp-1(e2141ts)III; fat-6(tm331)IV;* fat-5(tm420)V, made in the laboratory, HGA8010

*glp-1(e2141ts)III; fat-6(tm331)IV;* fat-7(wa36)V, made in the laboratory, HGA8011

*glp-1(e2141ts)III; daf-9(rh50)X;* LynEx, made in the laboratory, HGA8012

*glp-1(e2141ts)III; fat-6(tm331)IV; *fat-7(wa36)V;LynEx, made in the laboratory, HGA8013

*daf-2(e1370)III,* CGC, CB1370

* CGC = Caenorhabditis Genetics Center

** *lynEx = [(pJG01(nhr-80p::nhr-80::gfp)* and co-injection marker *myo-2p::DsRed]*

### Strain Construction

Double and triple mutant strains were generated using standard genetic procedures. The *glp-1(e2141ts)* mutation was assayed by testing sterility and lack of germ line at the restrictive temperature (25°C). Presence of the *nhr-80(tm1011), fat-5(tm420), fat-6(tm331)*, or *fat-7(wa36)* mutations were assayed by PCR using allele specific primers. To generate *nhr-80p::nhr-80::gfp* expressing animals (HGA8001; HGA8002; HGA8003; HGA8004; HGA8005; HGA8012; and HGA8013), *pJG01* was injected as described [28] at 50 ng/µL. Co-injection marker *myo-2p::DsRed* was injected at 20 ng/µL. Transgenes are called *lynEx*. The myo-2p::DsRed marker and *pPD95.75* (empty vector) have no effect on life span (unpublished data).

### *glp-1(e2141ts)* Suppressor Screening

RNAi clones from the Ahringer's Library were grown overnight at 37°C in LB containing Ampicillin (50 µg/mL) and Tetracyclin (12.5 µg/mL). Each RNAi clone was spread on NGM plates supplemented with carbenicillin (25 µg/mL). RNAi expression was induced by the addition of 1 mM IPTG on top of seeded bacteria. About 150 *glp-1(e2141ts)* (CF1903) and wild type L1 larvae were added on each RNAi plate and incubated at 25°C until day 1 of adulthood. At day 20 the proportion of worm alive was visually inspected. Clones that led to a majority of dead worms at this time for both wild type and *glp-1(e2141ts)* mutant animals were selected for further analysis.

### Plasmid Construction

The plasmid *pJG01(nhr-80p::nhr-80::gfp)* containing *nhr-80* tagged with GFP driven by the endogenous *nhr-80* promoter was constructed by amplifying genomic DNA from 1.4 kb upstream from the start codon until the end of the *nhr-80* coding sequence without the stop codon. The 4kb *XmaI*/*KpnI nhr-80* fragment generated was inserted upstream and in frame of the GFP sequence in the worm expression vector *pPD95.75* (Fire Lab Vector Kit–Addgene). Essential parts of the plasmid *pJG01* were sequenced.

Primer sequences:

Nhr80-XmaI-F: 5′-GGGGTGCC*CCCGGG*GGGA**TCGAGACACTTTTCTTACTCCTC**-3′

Nhr80-KpnI-R: 5′-AACGG*GGTACC*CCG**TTTTTCAAGCTTTGCCTGACCCA**-3′

### Lifespan Analyses

Lifespan assays were conducted according to standard protocols [29]. All assays were performed at 20°C, starting from day 1 of adulthood. For *glp-1(e2141ts)* mutants, lifespan assays (and associated controls), animals were grown at 25°C from the L1 stage until the L4 stage to prevent germ cell proliferation. The rest of the assay was performed at 20°C. For *cyc-1* RNAi experiments (and associated controls), larvae were left at 15°C for 24 h, shifted to 25°C for 24 h, and shifted back to 20°C at the L4 stage to avoid dauer formation or other larval arrest. All strains containing the *glp-1(e2141ts)* allele used in lifespan assays were completely sterile. Unless mentioned otherwise, lifespan assays of fertile strains were conducted on plates supplemented with 15 µM 5-Fluoro-Uracil in order to prevent progeny from hatching. Worms crawling off the plate, exploding, bagging, or contaminated were excluded. Plotting and statistical analysis were done using the Biopylife software. Biopylife was designed by students from INSA de Lyon using the following free softwares: R, MySQL, Python, MySQLdb, Qt4, PyQt4, and MacTeX. Biopylife allows easy plotting of lifespan assays and determines mean, maximal lifespan, and *p* values using log-rank (Mantel-Cox) statistics.

### Oleic Acid Supplementation

NGM medium were prepared with the addition of 100 µM oleic acid (NuChek Prep) right before pooring plates.

### Δ7 Dafachronic Acid Supplementation

Δ7 dafachronic acid was added on top of seeded bacteria to make a final NGM concentration of 100 nM. Worms were transferred on fresh plates every other day.

### Dietary Restriction 

DR was performed through bacterial deprivation from the fifth day of adulthood [30].

### qRT-PCR

For each gene, analyses were performed on triplicate of at least five independent extracts. For each analysis, we present two statistical tests: the parametric unpaired two-tailed *t* student test and the non-parametric Wilcoxon rank test. While the former test assumes a Gaussian distribution of the samples, the latter does not. Asterisk indicates the *p* value of the Wilcoxon rank-sum test as follows: *p<0.1, **p<0.05, ***p<0.01. Standard deviations are displayed as error bars.

#### RNA extraction and purification

Total RNA was isolated from synchronized populations of day-2 adult worms (about 3,000 individuals per condition) using the following method. Worms were harvested and washed three times with M9 buffer and twice with DEPC water. TRIZOL reagent (MRC) was added to the worm pellet (Trizol/worm pellet ratio was 2/1) and the mixture was vigorously shaken for at least 1 min. The mixture was frozen at −80°C overnight or for a longer period prior to the next RNA extraction steps.

Frozen worms were then placed on ice, vortexed 5 min, and settled at room temperature. The trizol/worm mix was transferred to eppendorf tubes and chloroform (200 µL per 1 mL trizol/worm mix) was added to each tube. Tubes were shaken for 15 s, incubated at room temperature 2 min, and centrifuged at 12,000 g for 5 min. The upper aqueous phase was transferred in a new tube. An equal volume of 70% ethanol (prepared in DEPC water) was added to the aqueous phase and tubes were gently mixed by inversion.

RNA extraction was then performed following the Rneasy (Qiagen) kit instructions, including the optional step of *DNAseI* digestion on column. RNAs were eluted in 50 µL RNAse free water. RNA concentrations were determined using a nanodrop spectrophotometer. RNA extracts were used directly or kept at −80°C.

#### RNA reverse transcription

cDNAs were created using the iScript cDNA Synthesis kit (Bio-Rad) using 1 ng RNA.

#### Quantitative real time PCR

SybrGreen real-time Q-PCR experiments were performed as described in the LightCycler FastStart DNA MasterPlus SYBR Green I manual.

#### Components for a reaction (slightly modified)

(Water PCR grade 6 µL, PCR primers 1 µL, Master Mix 2 µL)

Each pre-cooled capillary was filled with 9 µL of Q-PCR mix and 1 µL of cDNA template at a 1/10 dilution (or 1/10, 1/40, 1/160, 1/640, and 1/2,560 dilutions for standards). Quantitative qRT-PCR reactions were carried out on a Light Cycler 1.5 (Roche). Data were collected using RNA from at least three independent *C. elegans* cultures. Standard curve method was used to determine the relationship between mRNA abundance and PCR cycle number. Each primer sets were calibrated using serial dilutions of cDNA preparations. Primer sets were also calibrated by performing qRT-PCR reactions on serial dilutions of *C. elegans* genomic DNA. mRNA levels of the actin gene *act-1* were used for normalization.

##### Primer sequences

ACT-1-L, 5′-CCCACTCAATCCAAAGGCTA-3′

ACT-1-R, 5′-ATCTCCAGAGTCGAGGACGA-3′

DAF-16-L, 5′-TACGAATGGATGGTCCAGAA-3′

DAF-16-R, 5′-TCGCATGAAACGAGAATGAA-3′

DAF-12-L, 5′- CAACGCCCACTAACAATCAA -3′

DAF-12-R, 5′- GATACGGTTGTGCTCCTGGT -3′

FAT-5-L, 5′-TGAACTGGACCCGAGTATTGA-3′

FAT-5-R, 5′-ACAGCCGAACTTCTTGCACT-3′

FAT-6-L, 5′-GTCTCTGGTCCCACAAATCA-3′

FAT-6-R, 5′-TGGATCAGCATCGGTATCAG-3′

FAT-7-L, 5′-GGAAGGAGACAGCATTCATTGCG-3′

FAT-7-R, 5′-GTCTTGTGGGAATGTGTGGTGG-3′

NHR-80-L, 5′-TGAGGTTCAGGAGCCAAATAG-3′

NHR-80-R, 5′-GAAGGAGGTGGACGATGAGA-3′

K04A8.5-L, 5′-ATGGCCGAGAAGTTCCTACATCGT-3′

K04A8.5-R, 5′-GGTGAATTGGCGACCCAATCGAAA-3′

### DAF-16 Nuclear Localization Assays

DAF-16 nuclear localization assays were performed as previously described [4]. Briefly, *daf-16(mu86);glp-1(e2141ts);muIs109 [Pdaf-16::gfp::daf-16]* mutant animals were injected with water (negative control), *kri-1 RNAi* (control), or *nhr-80 RNAi* at 1 mg/mL and recovered at 15°C. The next day, animals were shifted to 20°C to lay eggs for several hours on OP50 plates. To obtain F1 animals with a *glp-1(e2141ts)* phenotype, eggs were shifted to 25°C, 24 h after being laid and shifted back to 20°C at the L4 stage. To obtain F1 animals without any *glp-1(e2141ts)* phenotype, eggs were left at 20°C during the whole course of the experiment. On day 1 of adulthood, F1 animals were assayed for DAF-16 nuclear localization in intestinal cells using a fluorescent microscope. Animals were scored as having nuclear-localized DAF-16 if the majority of intestinal cells displayed a distinct concentration of GFP in the nucleus. Twenty-five to 50 animals were analyzed for each condition.

### NHR-80::GFP Quantification

The MetaMorph software was used for image processing and ImageJ was used for fluorescence quantification. Fluorescence microscopy was performed using an axioplan microscope (Zeiss), with a cooled charge-coupled device camera. GFP images of sterile and fertile worms (i.e., grown until L4 at permissive or restrictive temperature and then switched at 20°C) were collected at day 1 of adulthood. Regions of Interest (ROI) were manually designed within either the two first intestinal nuclei and head neuronal nuclei; the software (ImageJ) calculated the mean intensity value for pixel intensity within the ROIs. At least 10 animals were analyzed for each condition. Averages and errors are presented in our graphs.

### Fatty Acid Methyl Ester Analysis

Each condition was analyzed in three independent extracts. For each extract, 4,000 to 5,000 adult worms at day 1 of adulthood grown at 25°C on ht115 were washed three times in M9 buffer. Worms were then homogenized in 2 ml of methanol/ 5 mM EGTA (2∶1 v/v) with FAST-PREP (MP Biochemicals). 100 ml were evaporated and the dry pellets were dissolved in 0.2 ml of NaOH (0.1 M) overnight and proteins were measured with the Bio-Rad assay. Lipids corresponding to the total homogenate were extracted according to Bligh and Dyer [31] in chloroform/methanol/water (2.5/2.5/2.1, v/v/v), in the presence of the internal standards glyceryl triheptadecanoate (5 g). The lipid extracts were hydrolysed in KOH (0.5 M in methanol) at 50°C for 30 min, and transmethylated in boron trifluoride methanol solution 14% (SIGMA, 1 ml) and hexane (1 ml) at 80°C for 1 h. After addition of water (1 ml) to the crude extract, FAMEs were extracted with hexane (3 ml), evaporated to dryness, and then dissolved in ethyl acetate (180 ml). FAME (1 ml) were analyzed by gas-liquid chromatography [32] on a Clarus 600 Perkin Elmer system using a Famewax RESTEK fused silica capillary columns (30 m×0.32 mm i.d., 0.25 m film thickness). Oven temperature was programmed from 110°C to 220°°C at a rate of 2°C per min and the carrier gas was hydrogen (0.5 bar). The injector and the detector were at 225°C and 245°C, respectively.

## Supporting Information

Figure S1 NHR-80::GFP is induced in intestinal cells when GSC proliferation is inhibited at different stages. Wild type and *glp-1(e2141ts)* mutant animals expressing a GFP tagged version of NHR-80 were used to monitor the induction of NHR-80 protein levels upon inhibition of GSC proliferation. The GFP levels in the two first intestinal cells were quantified after shifting animals to restrictive temperature (25°C) at different times. NHR-80::GFP levels do not change in neuronal cells (not shown). NHR-80::GFP levels are never affected in wild type animals while they are induced 1.6-fold, 1.4-fold, and 1.1-fold in *glp-1(e2141ts)* mutants when shifted at the L1, the L4, and at day 1 of adulthood, respectively. For the L1 and the L4 stages, the induction is statistical (Wilcoxon rank-sum test *p* value <0.01. *p<0.1, **p<0.05, ***p<0.01). For temperature shifts performed at day 1 of adulthood, the induction did not reach statistical significance (Wilcoxon rank-sum test *p* value not significant). Thus, NHR-80::GFP induction levels correlate with the extent of lifespan extension [2].
(5.72 MB TIF)

Figure S2 RNAi against *nhr-80* and a *loss-of-function* mutation to suppress *glp-1* longevity as efficiently. Knocking down *nhr-80* by RNAi suppresses *glp-1(e2141ts)* longevity as efficiently as the *nhr-80(tm1011)* mutation (mean lifespan of 12 d for *glp-1(e2141ts);nhr-80(tm1011)* and *glp-1(e2141ts)* on *nhr-80* RNAi; *p* = 0.82 when compared to one another). *nhr-80* RNAi and the *nhr-80(tm1011)* allele reduce the mean lifespan of *glp-1(e2141ts)* animals by 53% (*p*<0.0001). Lifespan analyses were performed at least twice independently. The *p* values were calculated using the log rank (Mantel-Cox) analyses. Because neurons are refractory to RNAi, this experiment also suggests that NHR-80 does not function in the neurons, but rather in the intestine (the other tissue where *nhr-80* is expressed).
(5.78 MB TIF)

Figure S3 The NHR-80::GFP transgene is functional. Transforming *glp-1(e2141ts);nhr-80(tm1011)* double mutants with the *nhr-80* transgene (*lynEx[(nhr-80p::nhr-80::gfp) myo-2p::DsRed]*) increases its mean lifespan by 70%. The resulting mean lifespan is similar to that of *glp-1(e2141ts)* single mutant animals (*p* = 0.46 between *glp-1(e2141ts)* and *glp-1(e2141ts);nhr-80(tm1011);lynEx[(nhr-80p::nhr-80::gfp) myo-2p::DsRed]*). Lifespan analyses were performed at least twice independently. The *p* values were calculated using the log rank (Mantel-Cox) analyses.
(5.95 MB TIF)

Figure S4 *nhr-80* RNAi further reduces the lifespan of *daf-16(mu86);glp-1(e2141ts)* double mutants. Lifespan analyses of *daf-16(mu86);glp-1(e2141ts)* treated with the empty vector (ev) or *nhr-80* RNAi (mean lifespan of 12 and 7 d, respectively; *p*<0.0001). Lifespan analyses were performed at least twice independently. The *p* values were calculated using the log rank (Mantel-Cox) analyses.
(5.66 MB TIF)

Figure S5 Lifespan analyses of *glp-1(e2141ts);daf-9(rh50)* mutants overexpressing *nhr-80* in the presence of Δ7 dafachronic. The overexpression of *nhr-80* extends the lifespan of *glp-1(e2141ts);daf-9(rh50)* mutants in the presence of Δ7 dafachronic acid (mean lifespan of 17 and 26 d for *glp-1(e2141ts);daf-9(rh50)* mutants overexpressing *nhr-80* and non-transgenic siblings, respectively; *p*<0.001). As controls, lifespan of *glp-1(e2141ts);daf-9(rh50)* and *glp-1(e2141ts)* mutant animals without addition Δ7 dafachronic are added. Lifespan analyses were performed at least twice independently. The *p* values were calculated using the log rank (Mantel-Cox) analyses.
(5.87 MB TIF)

Figure S6 DAF-12 binding sites on the NHR-80 promoter. Two distant DAF-12 binding half-sites (yellow and green) were found in the NHR-80 promoter. The presence of such binding sites on a promoter is not associated with elevated transcription levels of the associated gene [33]. In line with this, we found that mRNA levels of *nhr-80* are induced in *glp-1(e2141ts);daf-12(rh61rh411)* double mutants (Figure 5F).
(8.39 MB TIF)

Figure S7 Oleic acid levels and the oleic/stearic acid ratio are increased in *glp-1(e2141ts)* mutants in a *nhr-80* dependent manner. (A) Oleic acid levels are increased by 20% in *glp-1(e2141ts)* mutants compared to wild type animals (Wilcoxon rank-sum test *p* value <0.05). (B) Similarly, the Oleic/Stearic acid ratio is increased by 30% in *glp-1(e2141ts)* mutants compared to wild type animals (Wilcoxon rank-sum test *p* value <0.1). *nhr-80* deletion decreases this ratio, independently of the status of the germ line.
(6.16 MB TIF)

Figure S8 Deletion of individual *fat* genes does not affect the lifespan of *glp-1(e2141ts)* mutant animals. Lifespan analyses of *glp-1(e2141ts);fat-5(tm420)*, *glp-1(e2141ts);fat-6(tm331)*, and *glp-1(e2141ts);fat-7(wa36)* mutants (*p* = 0.35, *p* = 0.06, and *p* = 0.79 when compared to *glp-1(e2141ts)*, respectively). Lifespan analyses were performed at least twice independently. The *p* values were calculated using the log rank (Mantel-Cox) analyses.
(2.48 MB TIF)

Figure S9 *fat-7* mRNA levels are induced in the absence of *fat-6* in germline-less animals. *fat-7* mRNA levels are reduced 5.5-fold in *glp-1(e2141ts)* compared to wild type animals (5.5-fold decrease Wilcoxon rank-sum test *p* value <0.001; error bars are standard deviation, *p<0.1, **p<0.05, ***p<0.01). However, in the absence of *fat-6*, *fat-7* mRNA levels are strongly up-regulated (358-fold induction; Wilcoxon rank-sum test *p*<0.01; error bars are standard deviation, *p<0.1, **p<0.05, ***p<0.01). This compensatory mechanism has already been described in wild type animals [18]. This likely accounts for the normal longevity observed in *glp-1(e2141ts);fat-6(tm331)* animals.
(5.75 MB TIF)

Figure S10 Effect of the double mutations *fat-7(wa36);fat-5(tm420)* and *fat-6(tm331);fat-5(tm420)* on lifespan. (A) Lifespan analyses of *glp-1(e2141ts)*, *glp-1(e2141ts);fat-7(wa36);fat-5(tm420)*, and *glp-1(e2141ts);fat-6(tm331);fat-5(tm420)* (mean lifespan of 26, 28, and 20 d, respectively). (B) Lifespan analyses of wild type, *fat-7(wa36);fat-5(tm420)*, *fat-6(tm331);fat-7(wa36)*, and *fat-6(tm331);fat-5(tm420)*. The lifespan of these animals are similar to that of wild type animals (mean lifespan of 19, 17, and 15.5 d, respectively; *p* = 0.01, 0.2, and 0.21 when compared to the wild type, respectively). (C) Lifespan analyses of wild type, *fat-7(wa36);fat-5(tm420)*, *fat-6(tm331);fat-7(wa36)*, and *fat-6(tm331);fat-5(tm420)* in the presence of oleic acid. Oleic acid does not modify the lifespan of these animals (mean lifespan of 17, 15.5, and 15.5 d, respectively; *p* = 0.01, 0.12, and 0.09 when compared to treated wild type, respectively). Lifespan analyses were performed at least twice independently. The *p* values were calculated using the log rank (Mantel-Cox) analyses.
(6.08 MB TIF)

Figure S11 Effect of the deactivation of *fat-2* on lifespan and fertility. (A) Lifespan analyses of wild type and *glp-1(e2141ts)* mutants on either an empty vector or *fat-2* RNAi. *fat-2* encodes for a Δ12 fatty acyl desaturase that further desaturates OA to polyunsaturated fatty acids. *fat-2* RNAi did not affect the lifespan of either wild type (mean lifespan of 13 and 14.5 d for empty vector and *fat-2* RNAi, respectively; *p* = 0.13) or *glp-1(e2141ts)* mutants (mean lifespan of 29.5 and 27.5 d for empty vector and *fat-2* RNAi, respectively; *p* = 0.54). The *p* values were calculated using the log rank (Mantel-Cox) analyses. (B) *fat-2* RNAi is functional since it strongly altered egg laying of wild type animals as previously shown [34].
(6.24 MB TIF)

Figure S12 Oleic acid does not affect the lifespan of *daf-16(mu86);glp-1(e2141ts)*, *glp-1(e2141ts);daf-12(rh61rh411)*, and *glp-1(e2141ts);daf-9(rh50)* double mutants. Lifespan analyses of *daf-16(mu86);glp-1(e2141ts)*, *glp-1(e2141ts);daf-12(rh61rh411)*, and *glp-1(e2141ts);daf-9(rh50)* double mutants with or without oleic acid. For *daf-16(mu86);glp-1(e2141ts)*, the mean lifespan is of 11 d for both treated and untreated animals; *p* value  = 0.5 between treated and untreated animals. For *glp-1(e2141ts);daf-12(rh61rh411)*, treated and untreated animals have a mean lifespan of 19.5 and 16 d, respectively; *p* value  = 0.37 between treated and untreated animals. For *glp-1(e2141ts);daf-9(rh50)*, the mean lifespan is of 10.5 d for both treated and untreated animals, *p* value  = 0.43 between treated and untreated animals. Lifespan analyses were performed at least twice independently. The *p* values were calculated using the log rank (Mantel-Cox) analyses.
(5.80 MB TIF)

Figure S13 *fat-6* expression does not require the presence of *daf-12*. *fat-6* mRNA levels are increased in *glp-1(e2141ts)* and in *glp-1(e2141ts);daf-12(rh61rh411)* mutants as measured by qRT-PCR (5.5- and 4.4-fold increase; Wilcoxon rank-sum test *p* value <0.001 for both strains when compared with N2). When *glp-1(e2141ts);daf-12(rh61rh411)* mutants are compared to *glp-1(e2141ts)* mutants, there is an 0.8-fold decrease (Wilcoxon rank-sum test *p* value is not significant). Error bars are standard deviation. This suggests that *fat-6* mRNA levels respond to *glp-1(e2141ts)* mutants and that part of this response is *daf-12* independent. A control experiment shows that *fat-6* is not under the control of *daf-12* in the wild type background (Wilcoxon rank-sum test *p* value is non-significant).
(5.64 MB TIF)

Table S1 Summary of adult lifespan data presented in this work.
(0.05 MB PDF)

## Abbreviations

DR: dietary restriction
FA: fatty acid
FOXO: forkhead box O
GFP: green fluorescent protein
GSC: germline stem cells
HNF4: hepatocyte nuclear factor 4
IGF1: insulin-like growth factor 1
IIS: insulin/igf1 signaling
L1: larval stage 1
L4: larval stage 4
MLS: mean lifespan
NHR: nuclear hormone receptor
OA: oleic acid
PCD: Palmitoyl-CoA-Δ9-Desaturase
qRT-PCR: quantitative real time reverse transcription PCR
RNAi: RNA interference
SCD: Stearoyl-CoA-Δ9-Desaturase
TAG: triacylglycerols
VDR: vitamin D receptor
