# Supplementary figures and images for: Fatty Acid Desaturation Links Germ Cell Loss to Longevity Through NHR-80/HNF4 in C. elegans
Source: PLoS Biol. 2011 Mar 15;9(3):e1000599. doi: 10.1371/journal.pbio.1000599 (PMC3057950; doi:10.1371/journal.pbio.1000599)

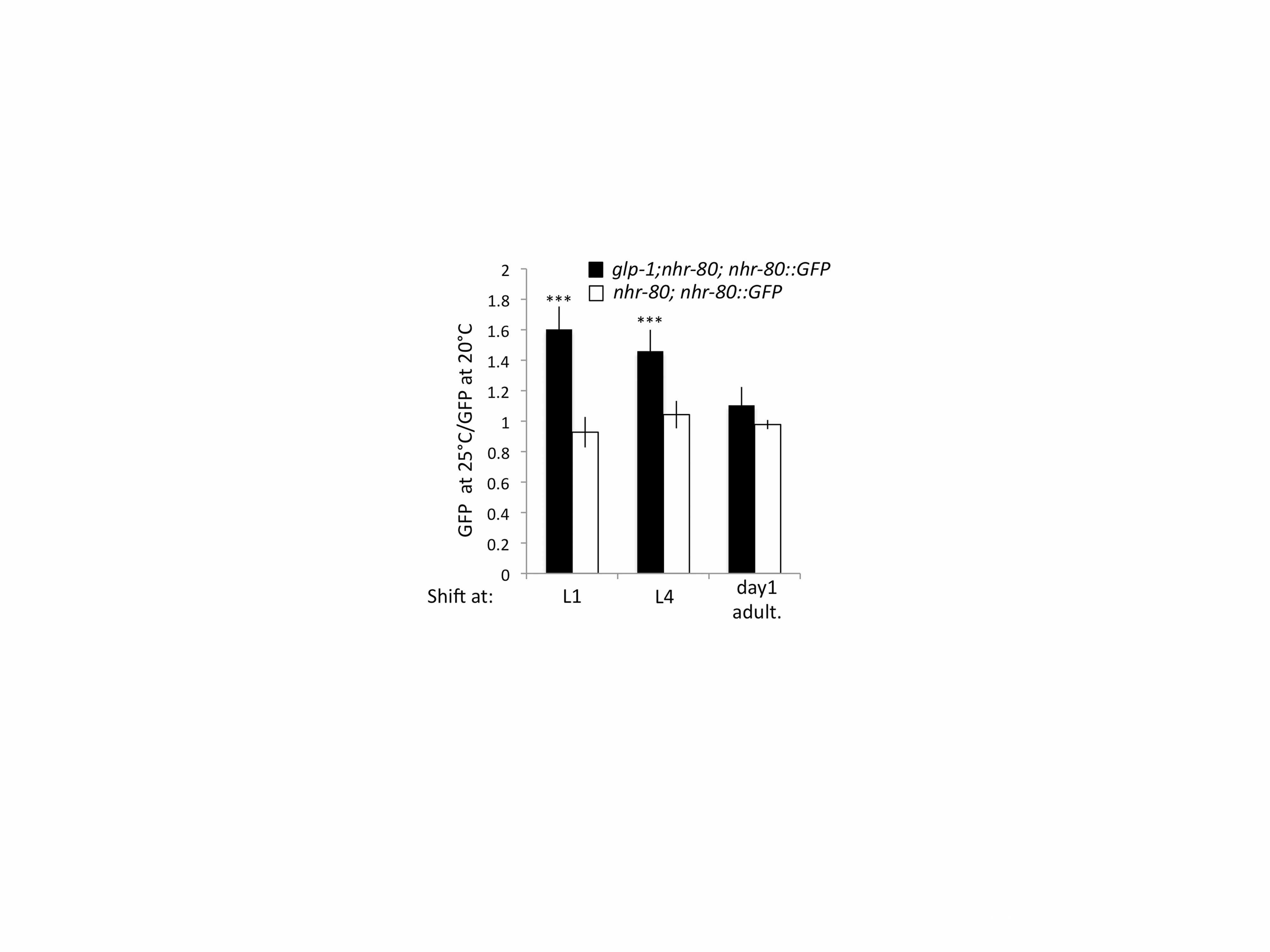

Supplement: Figure S1 — NHR-80::GFP is induced in intestinal cells when GSC proliferation is inhibited at different stages. Wild type and glp-1(e2141ts) mutant animals expressing a GFP tagged version of NHR-80 were used to monitor the induction of NHR-80 protein levels upon inhibition of GSC proliferation. The GFP levels in the two first intestinal cells were quantified after shifting animals to restrictive temperature (25°C) at different times. NHR-80::GFP levels do not change in neuronal cells (not shown). NHR-80::GFP levels are never affected in wild type animals while they are induced 1.6-fold, 1.4-fold, and 1.1-fold in glp-1(e2141ts) mutants when shifted at the L1, the L4, and at day 1 of adulthood, respectively. For the L1 and the L4 stages, the induction is statistical (Wilcoxon rank-sum test p value <0.01. *p<0.1, **p<0.05, ***p<0.01). For temperature shifts performed at day 1 of adulthood, the induction did not reach statistical significance (Wilcoxon rank-sum test p value not significant). Thus, NHR-80::GFP induction levels correlate with the extent of lifespan extension [2]. (5.72 MB TIF) [file pbio.1000599.s001.tif]

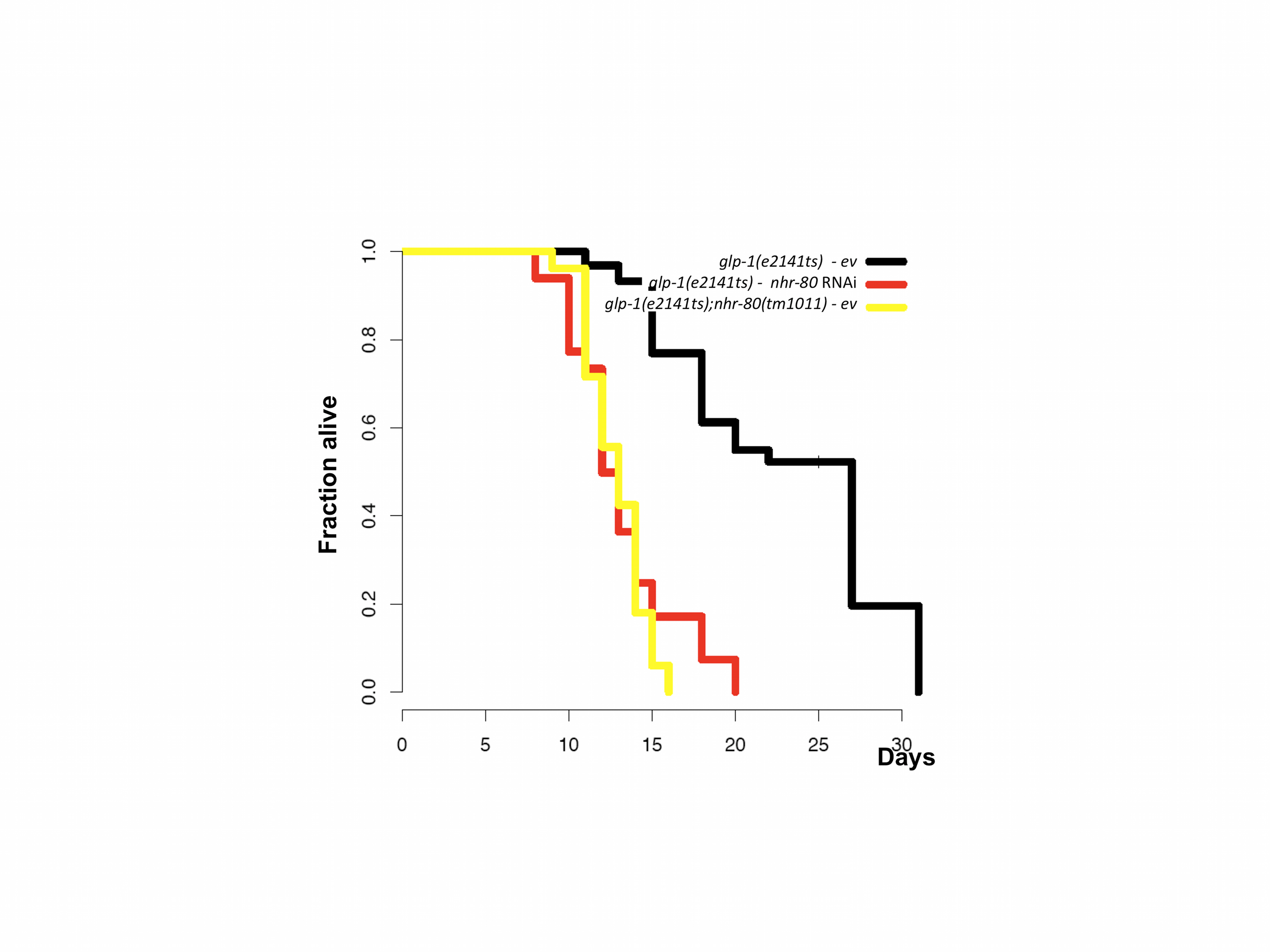

Supplement: Figure S2 — RNAi against nhr-80 and a loss-of-function mutation to suppress glp-1 longevity as efficiently. Knocking down nhr-80 by RNAi suppresses glp-1(e2141ts) longevity as efficiently as the nhr-80(tm1011) mutation (mean lifespan of 12 d for glp-1(e2141ts);nhr-80(tm1011) and glp-1(e2141ts) on nhr-80 RNAi; p = 0.82 when compared to one another). nhr-80 RNAi and the nhr-80(tm1011) allele reduce the mean lifespan of glp-1(e2141ts) animals by 53% (p<0.0001). Lifespan analyses were performed at least twice independently. The p values were calculated using the log rank (Mantel-Cox) analyses. Because neurons are refractory to RNAi, this experiment also suggests that NHR-80 does not function in the neurons, but rather in the intestine (the other tissue where nhr-80 is expressed). (5.78 MB TIF) [file pbio.1000599.s002.tif]

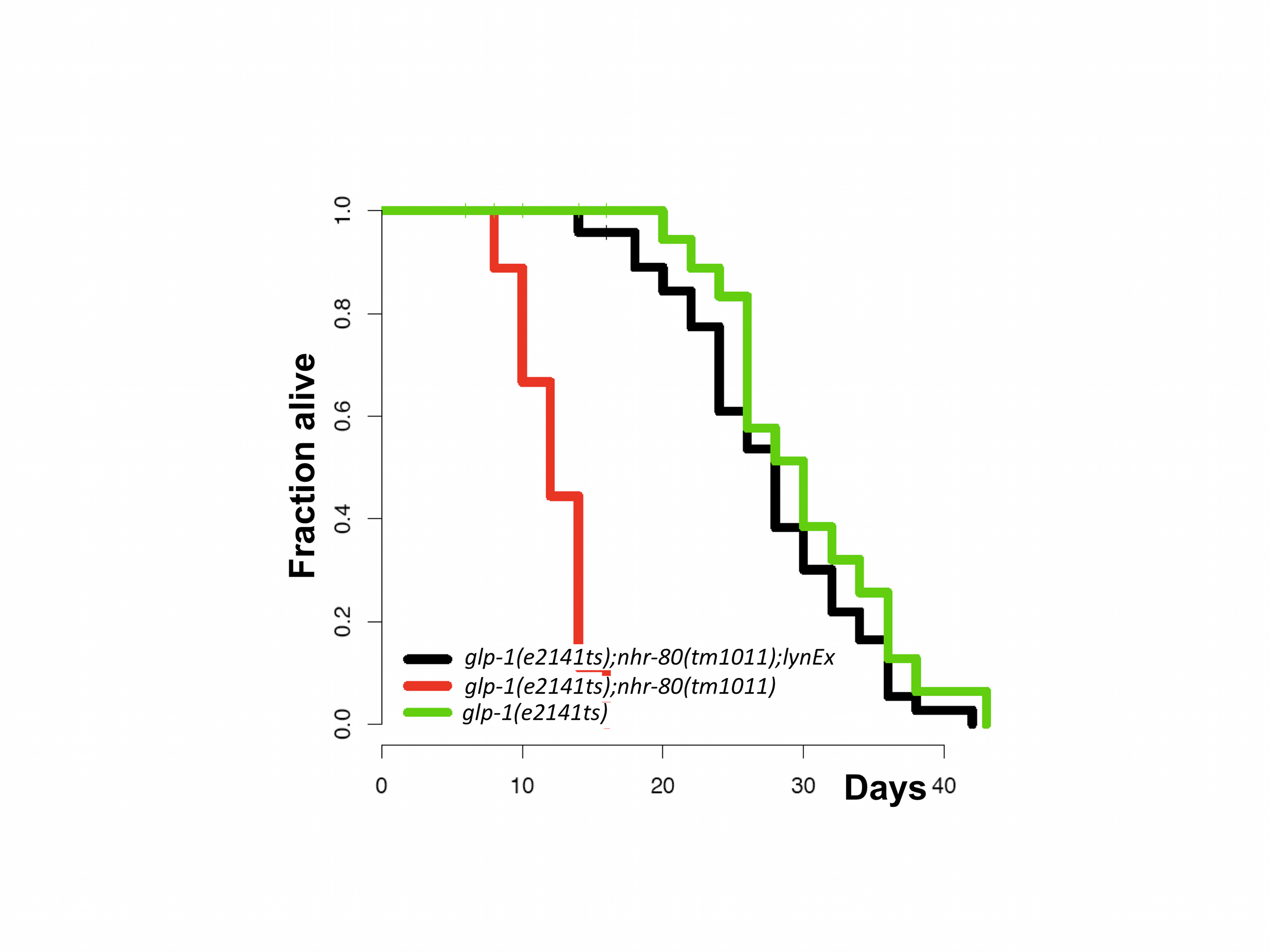

Supplement: Figure S3 — The NHR-80::GFP transgene is functional. Transforming glp-1(e2141ts);nhr-80(tm1011) double mutants with the nhr-80 transgene (lynEx[(nhr-80p::nhr-80::gfp) myo-2p::DsRed]) increases its mean lifespan by 70%. The resulting mean lifespan is similar to that of glp-1(e2141ts) single mutant animals (p = 0.46 between glp-1(e2141ts) and glp-1(e2141ts);nhr-80(tm1011);lynEx[(nhr-80p::nhr-80::gfp) myo-2p::DsRed]). Lifespan analyses were performed at least twice independently. The p values were calculated using the log rank (Mantel-Cox) analyses. (5.95 MB TIF) [file pbio.1000599.s003.tif]

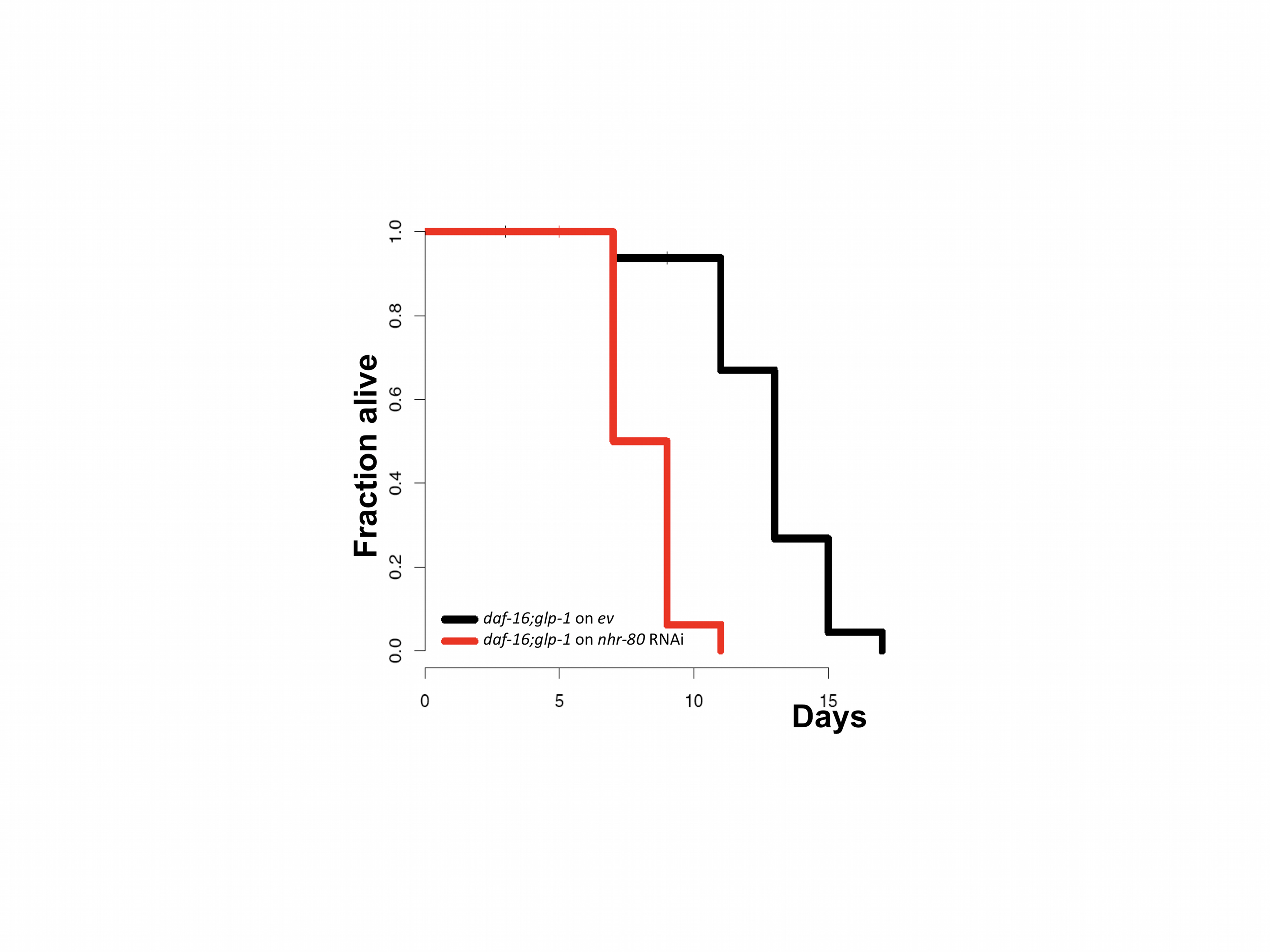

Supplement: Figure S4 — nhr-80 RNAi further reduces the lifespan of daf-16(mu86);glp-1(e2141ts) double mutants. Lifespan analyses of daf-16(mu86);glp-1(e2141ts) treated with the empty vector (ev) or nhr-80 RNAi (mean lifespan of 12 and 7 d, respectively; p<0.0001). Lifespan analyses were performed at least twice independently. The p values were calculated using the log rank (Mantel-Cox) analyses. (5.66 MB TIF) [file pbio.1000599.s004.tif]

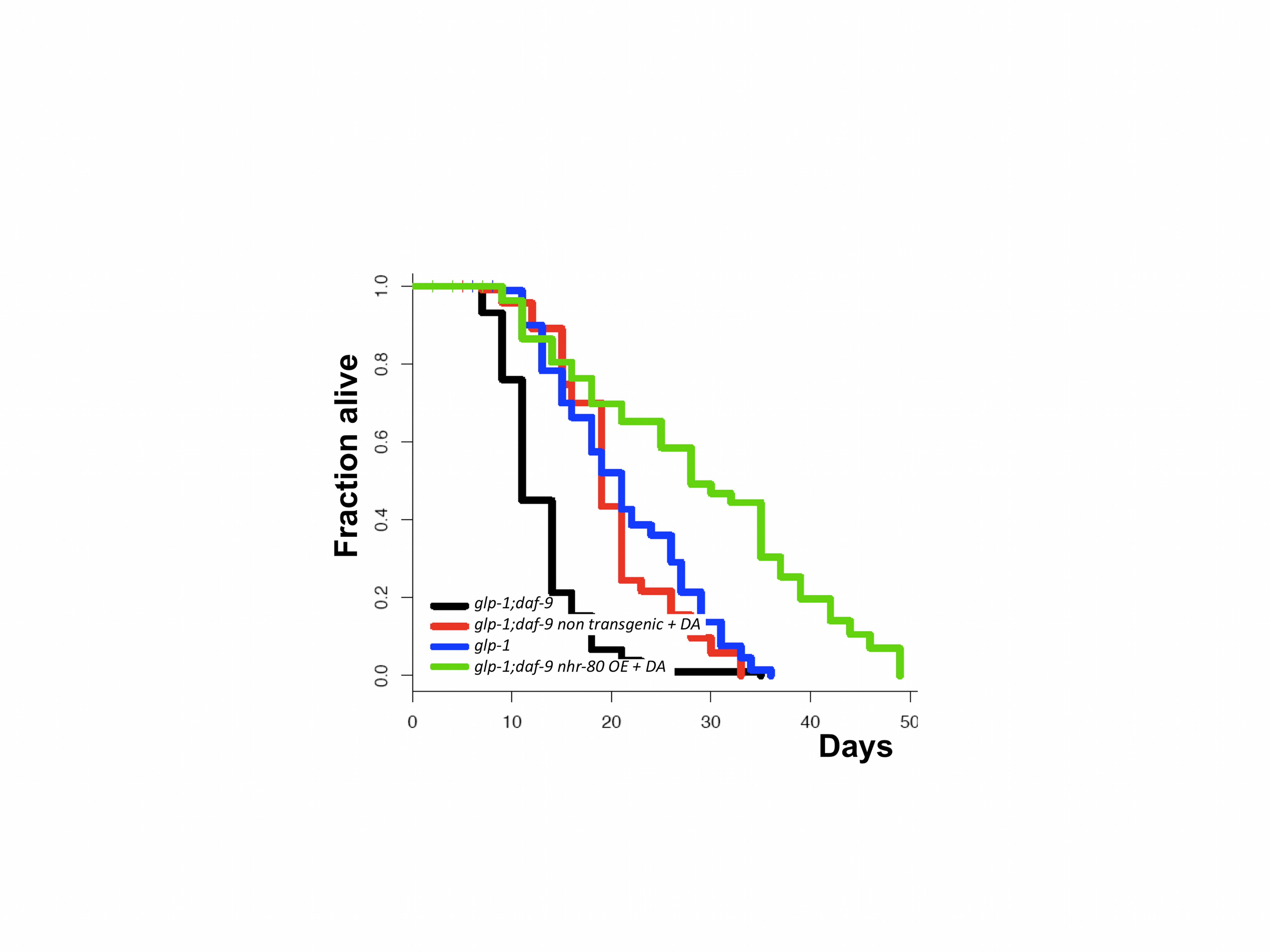

Supplement: Figure S5 — Lifespan analyses of glp-1(e2141ts);daf-9(rh50) mutants overexpressing nhr-80 in the presence of Δ7 dafachronic. The overexpression of nhr-80 extends the lifespan of glp-1(e2141ts);daf-9(rh50) mutants in the presence of Δ7 dafachronic acid (mean lifespan of 17 and 26 d for glp-1(e2141ts);daf-9(rh50) mutants overexpressing nhr-80 and non-transgenic siblings, respectively; p<0.001). As controls, lifespan of glp-1(e2141ts);daf-9(rh50) and glp-1(e2141ts) mutant animals without addition Δ7 dafachronic are added. Lifespan analyses were performed at least twice independently. The p values were calculated using the log rank (Mantel-Cox) analyses. (5.87 MB TIF) [file pbio.1000599.s005.tif]

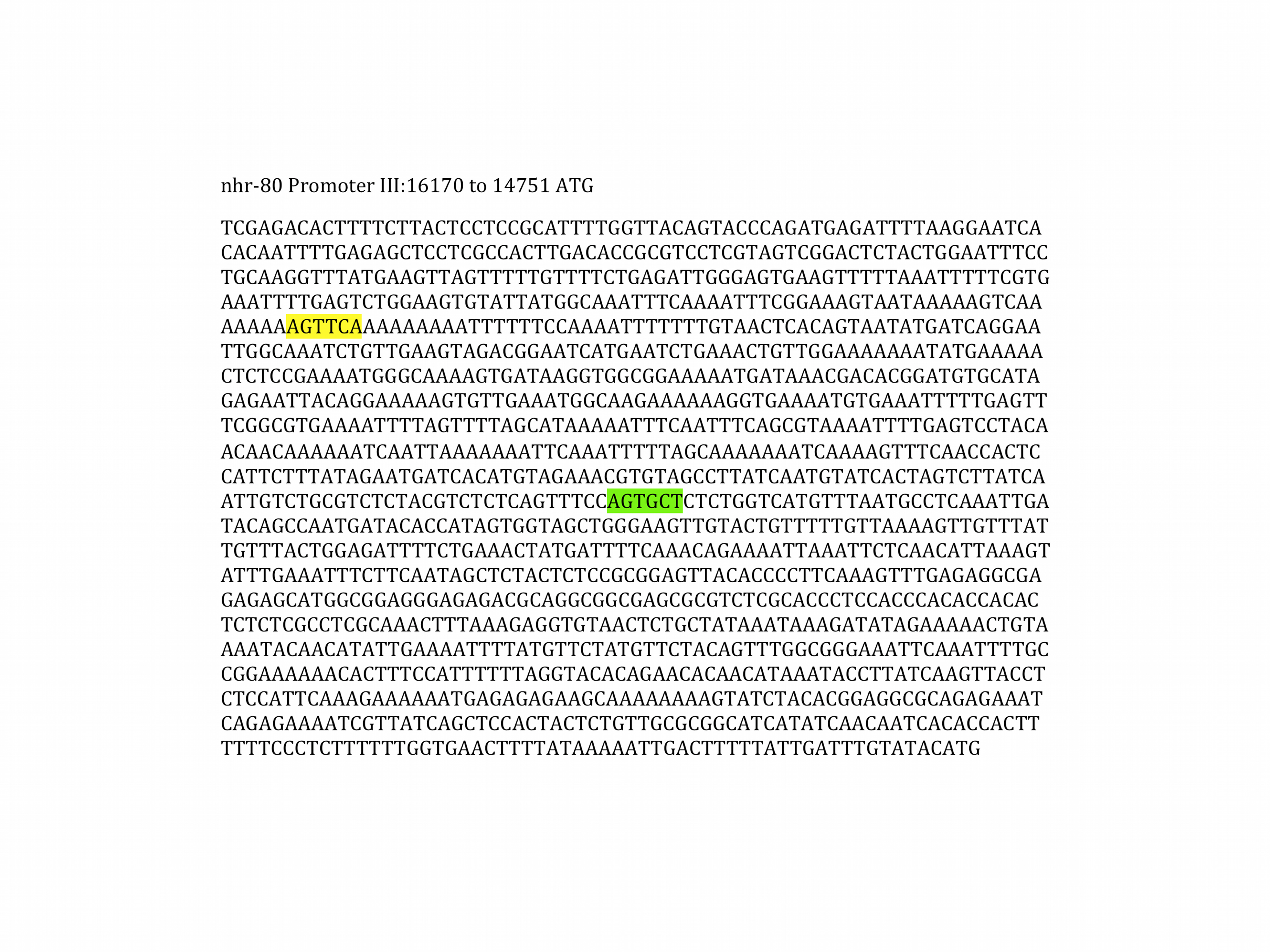

Supplement: Figure S6 — DAF-12 binding sites on the NHR-80 promoter. Two distant DAF-12 binding half-sites (yellow and green) were found in the NHR-80 promoter. The presence of such binding sites on a promoter is not associated with elevated transcription levels of the associated gene [33]. In line with this, we found that mRNA levels of nhr-80 are induced in glp-1(e2141ts);daf-12(rh61rh411) double mutants (Figure 5F). (8.39 MB TIF) [file pbio.1000599.s006.tif]

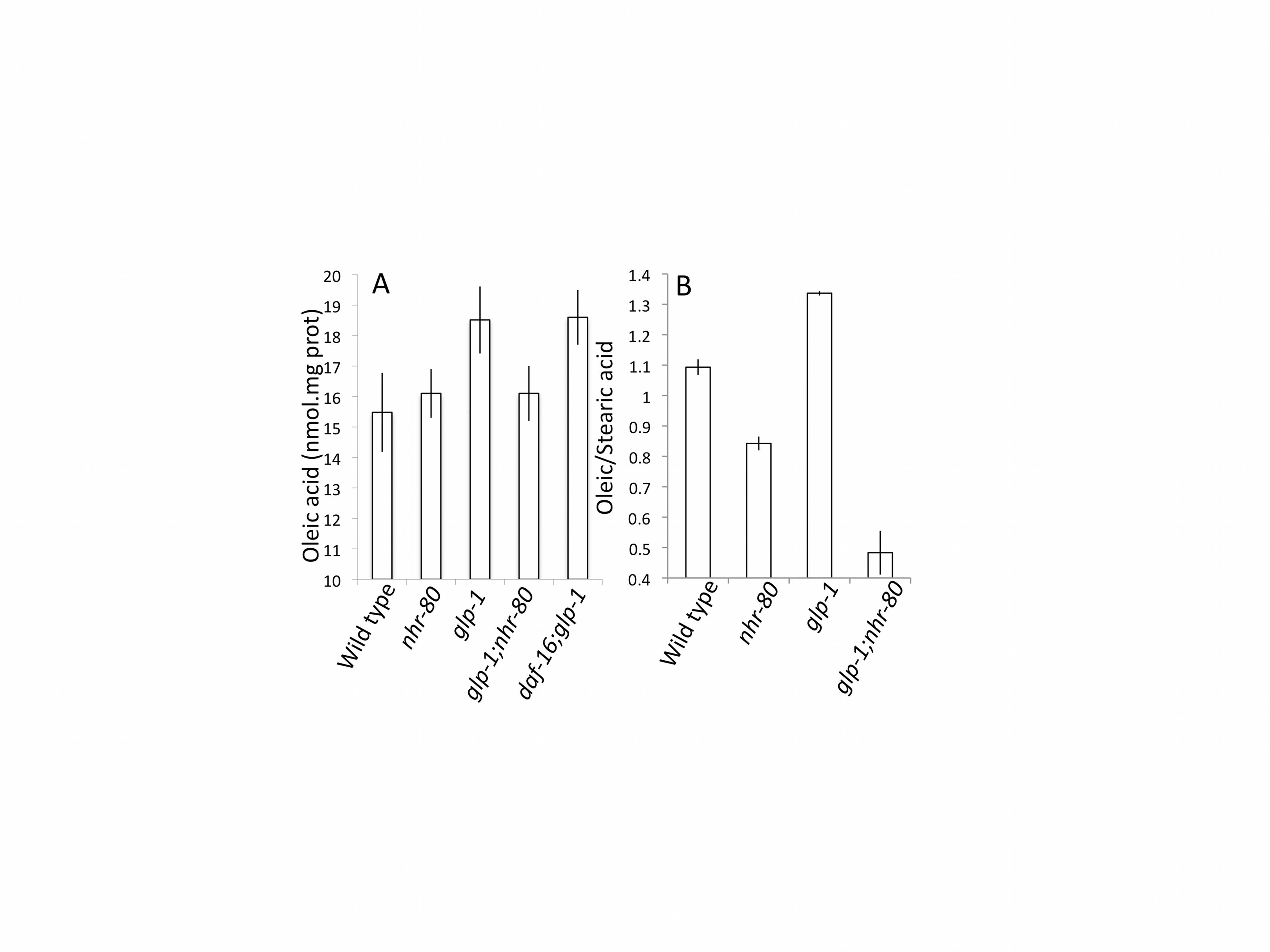

Supplement: Figure S7 — Oleic acid levels and the oleic/stearic acid ratio are increased in glp-1(e2141ts) mutants in a nhr-80 dependent manner. (A) Oleic acid levels are increased by 20% in glp-1(e2141ts) mutants compared to wild type animals (Wilcoxon rank-sum test p value <0.05). (B) Similarly, the Oleic/Stearic acid ratio is increased by 30% in glp-1(e2141ts) mutants compared to wild type animals (Wilcoxon rank-sum test p value <0.1). nhr-80 deletion decreases this ratio, independently of the status of the germ line. (6.16 MB TIF) [file pbio.1000599.s007.tif]

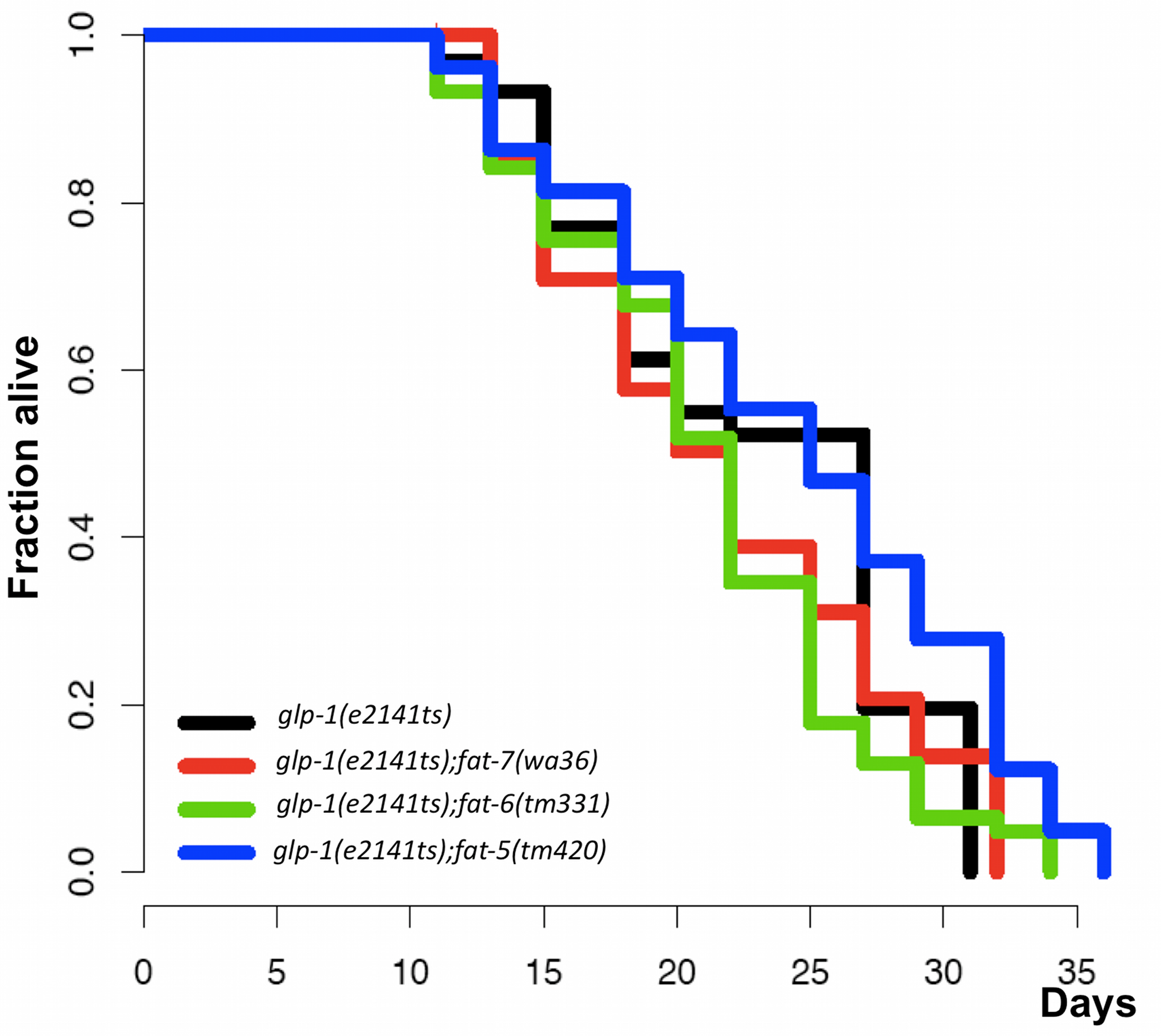

Supplement: Figure S8 — Deletion of individual fat genes does not affect the lifespan of glp-1(e2141ts) mutant animals. Lifespan analyses of glp-1(e2141ts);fat-5(tm420), glp-1(e2141ts);fat-6(tm331), and glp-1(e2141ts);fat-7(wa36) mutants (p = 0.35, p = 0.06, and p = 0.79 when compared to glp-1(e2141ts), respectively). Lifespan analyses were performed at least twice independently. The p values were calculated using the log rank (Mantel-Cox) analyses. (2.48 MB TIF) [file pbio.1000599.s008.tif]

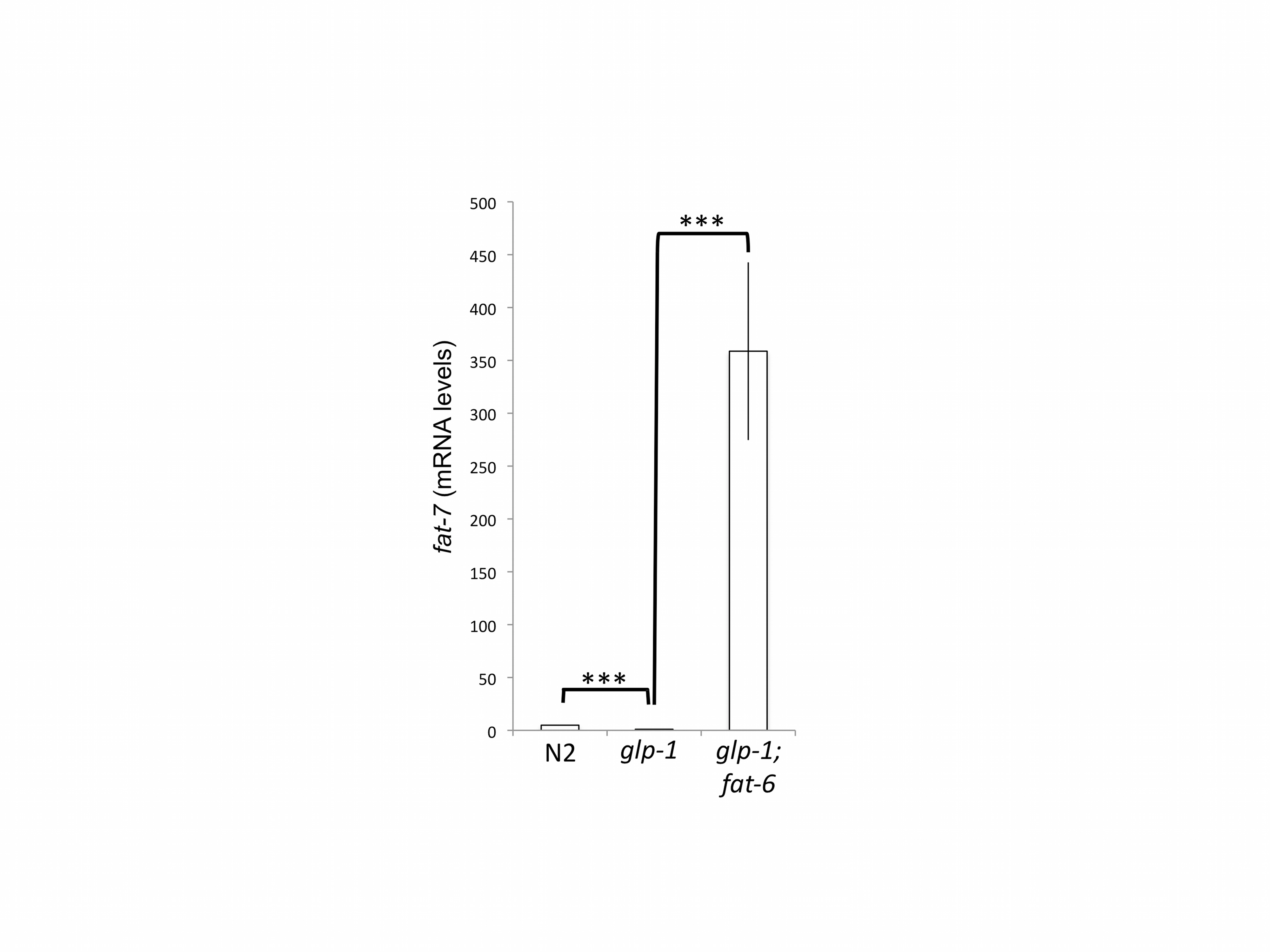

Supplement: Figure S9 — fat-7 mRNA levels are induced in the absence of fat-6 in germline-less animals. fat-7 mRNA levels are reduced 5.5-fold in glp-1(e2141ts) compared to wild type animals (5.5-fold decrease Wilcoxon rank-sum test p value <0.001; error bars are standard deviation, *p<0.1, **p<0.05, ***p<0.01). However, in the absence of fat-6, fat-7 mRNA levels are strongly up-regulated (358-fold induction; Wilcoxon rank-sum test p<0.01; error bars are standard deviation, *p<0.1, **p<0.05, ***p<0.01). This compensatory mechanism has already been described in wild type animals [18]. This likely accounts for the normal longevity observed in glp-1(e2141ts);fat-6(tm331) animals. (5.75 MB TIF) [file pbio.1000599.s009.tif]

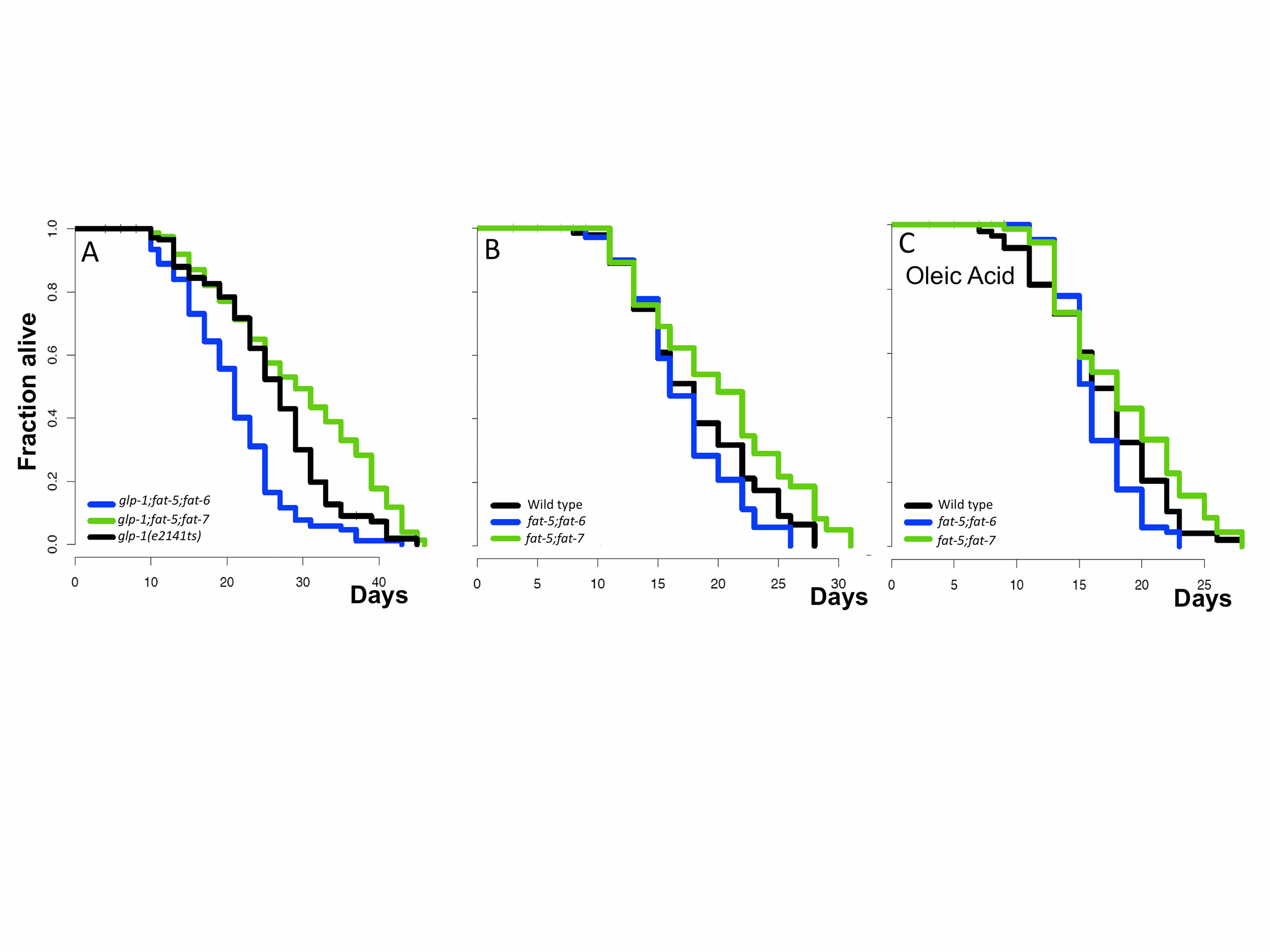

Supplement: Figure S10 — Effect of the double mutations fat-7(wa36);fat-5(tm420) and fat-6(tm331);fat-5(tm420) on lifespan. (A) Lifespan analyses of glp-1(e2141ts), glp-1(e2141ts);fat-7(wa36);fat-5(tm420), and glp-1(e2141ts);fat-6(tm331);fat-5(tm420) (mean lifespan of 26, 28, and 20 d, respectively). (B) Lifespan analyses of wild type, fat-7(wa36);fat-5(tm420), fat-6(tm331);fat-7(wa36), and fat-6(tm331);fat-5(tm420). The lifespan of these animals are similar to that of wild type animals (mean lifespan of 19, 17, and 15.5 d, respectively; p = 0.01, 0.2, and 0.21 when compared to the wild type, respectively). (C) Lifespan analyses of wild type, fat-7(wa36);fat-5(tm420), fat-6(tm331);fat-7(wa36), and fat-6(tm331);fat-5(tm420) in the presence of oleic acid. Oleic acid does not modify the lifespan of these animals (mean lifespan of 17, 15.5, and 15.5 d, respectively; p = 0.01, 0.12, and 0.09 when compared to treated wild type, respectively). Lifespan analyses were performed at least twice independently. The p values were calculated using the log rank (Mantel-Cox) analyses. (6.08 MB TIF) [file pbio.1000599.s010.tif]

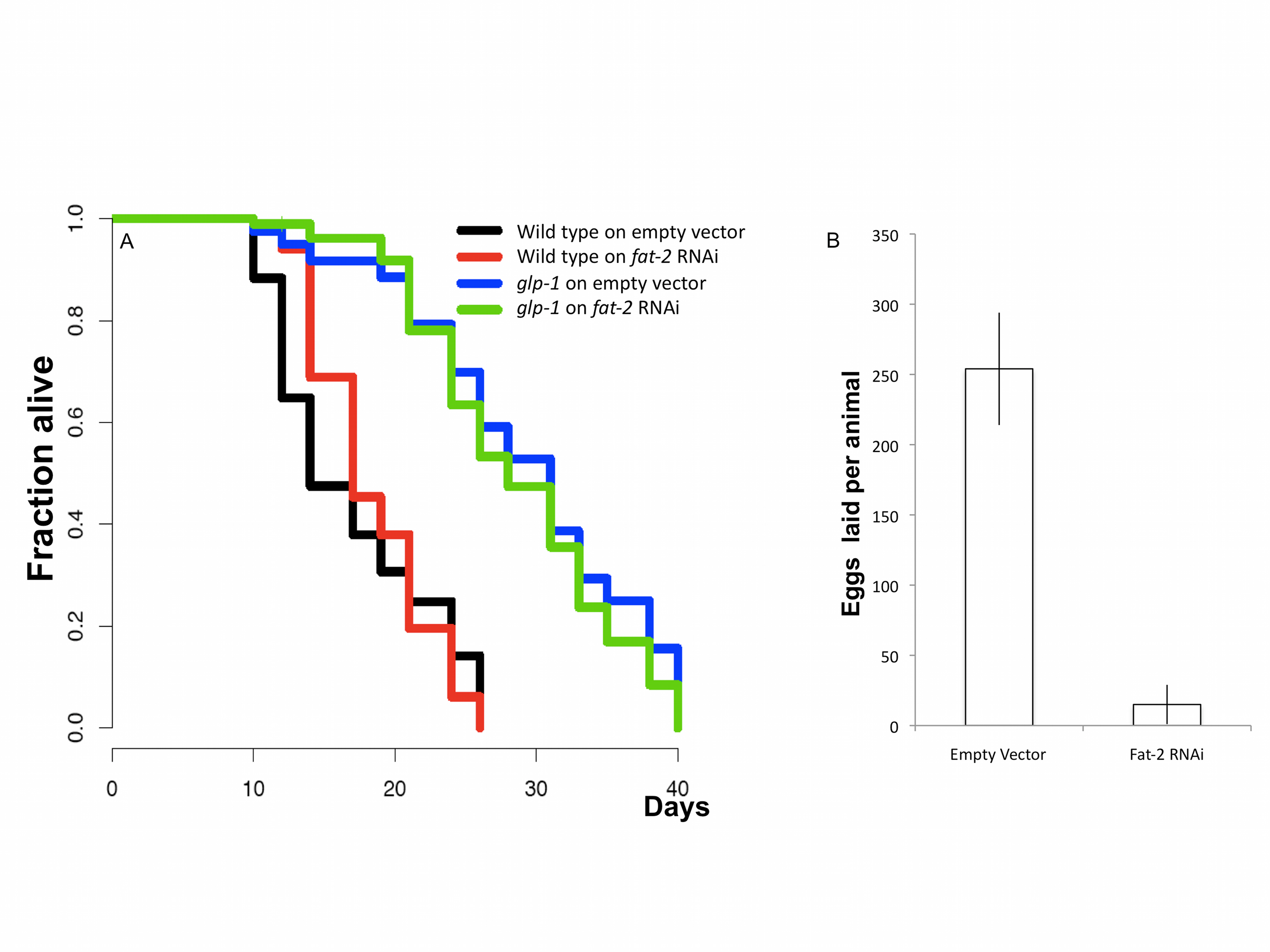

Supplement: Figure S11 — Effect of the deactivation of fat-2 on lifespan and fertility. (A) Lifespan analyses of wild type and glp-1(e2141ts) mutants on either an empty vector or fat-2 RNAi. fat-2 encodes for a Δ12 fatty acyl desaturase that further desaturates OA to polyunsaturated fatty acids. fat-2 RNAi did not affect the lifespan of either wild type (mean lifespan of 13 and 14.5 d for empty vector and fat-2 RNAi, respectively; p = 0.13) or glp-1(e2141ts) mutants (mean lifespan of 29.5 and 27.5 d for empty vector and fat-2 RNAi, respectively; p = 0.54). The p values were calculated using the log rank (Mantel-Cox) analyses. (B) fat-2 RNAi is functional since it strongly altered egg laying of wild type animals as previously shown [34]. (6.24 MB TIF) [file pbio.1000599.s011.tif]

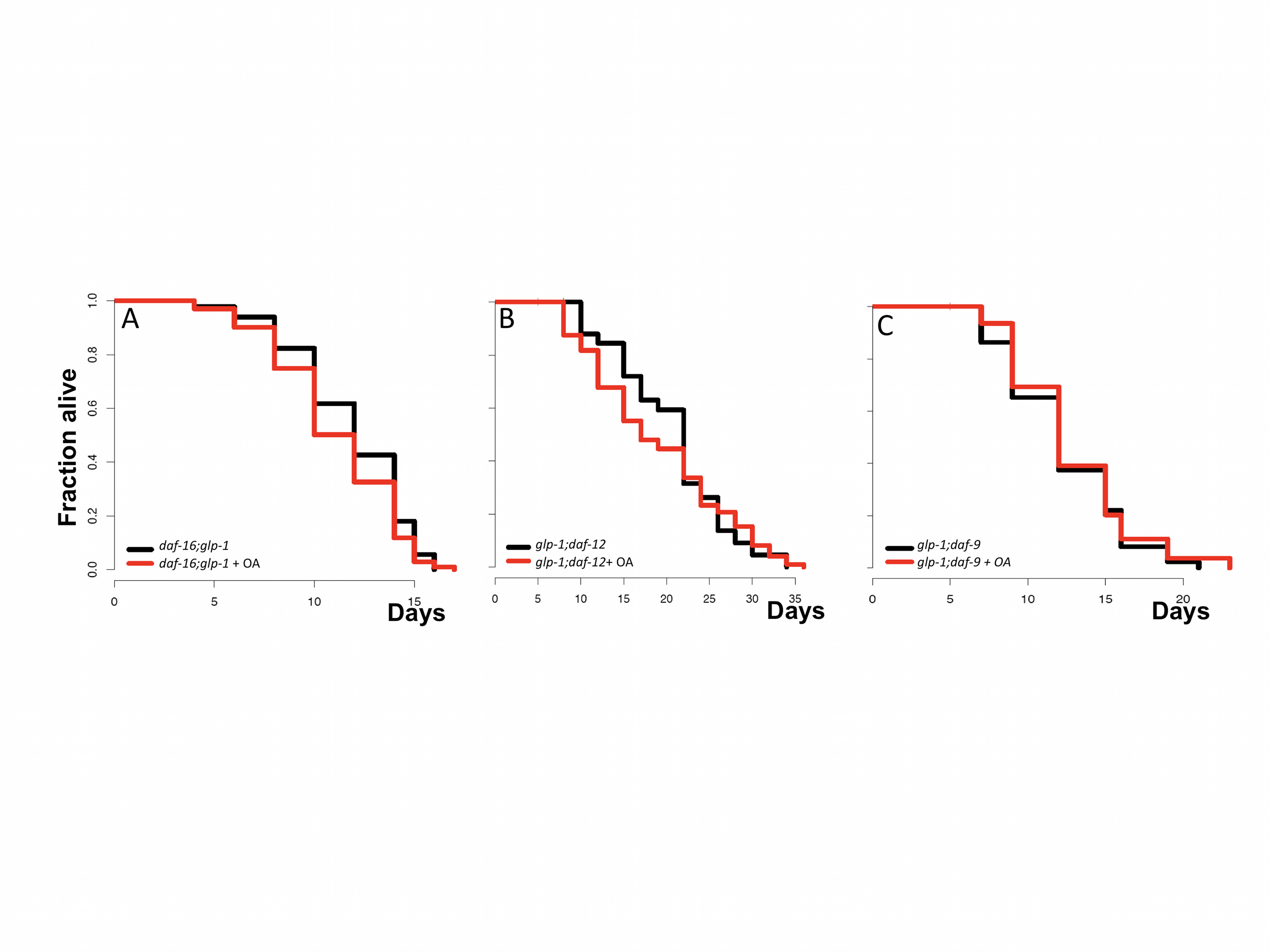

Supplement: Figure S12 — Oleic acid does not affect the lifespan of daf-16(mu86);glp-1(e2141ts), glp-1(e2141ts);daf-12(rh61rh411), and glp-1(e2141ts);daf-9(rh50) double mutants. Lifespan analyses of daf-16(mu86);glp-1(e2141ts), glp-1(e2141ts);daf-12(rh61rh411), and glp-1(e2141ts);daf-9(rh50) double mutants with or without oleic acid. For daf-16(mu86);glp-1(e2141ts), the mean lifespan is of 11 d for both treated and untreated animals; p value = 0.5 between treated and untreated animals. For glp-1(e2141ts);daf-12(rh61rh411), treated and untreated animals have a mean lifespan of 19.5 and 16 d, respectively; p value = 0.37 between treated and untreated animals. For glp-1(e2141ts);daf-9(rh50), the mean lifespan is of 10.5 d for both treated and untreated animals, p value = 0.43 between treated and untreated animals. Lifespan analyses were performed at least twice independently. The p values were calculated using the log rank (Mantel-Cox) analyses. (5.80 MB TIF) [file pbio.1000599.s012.tif]

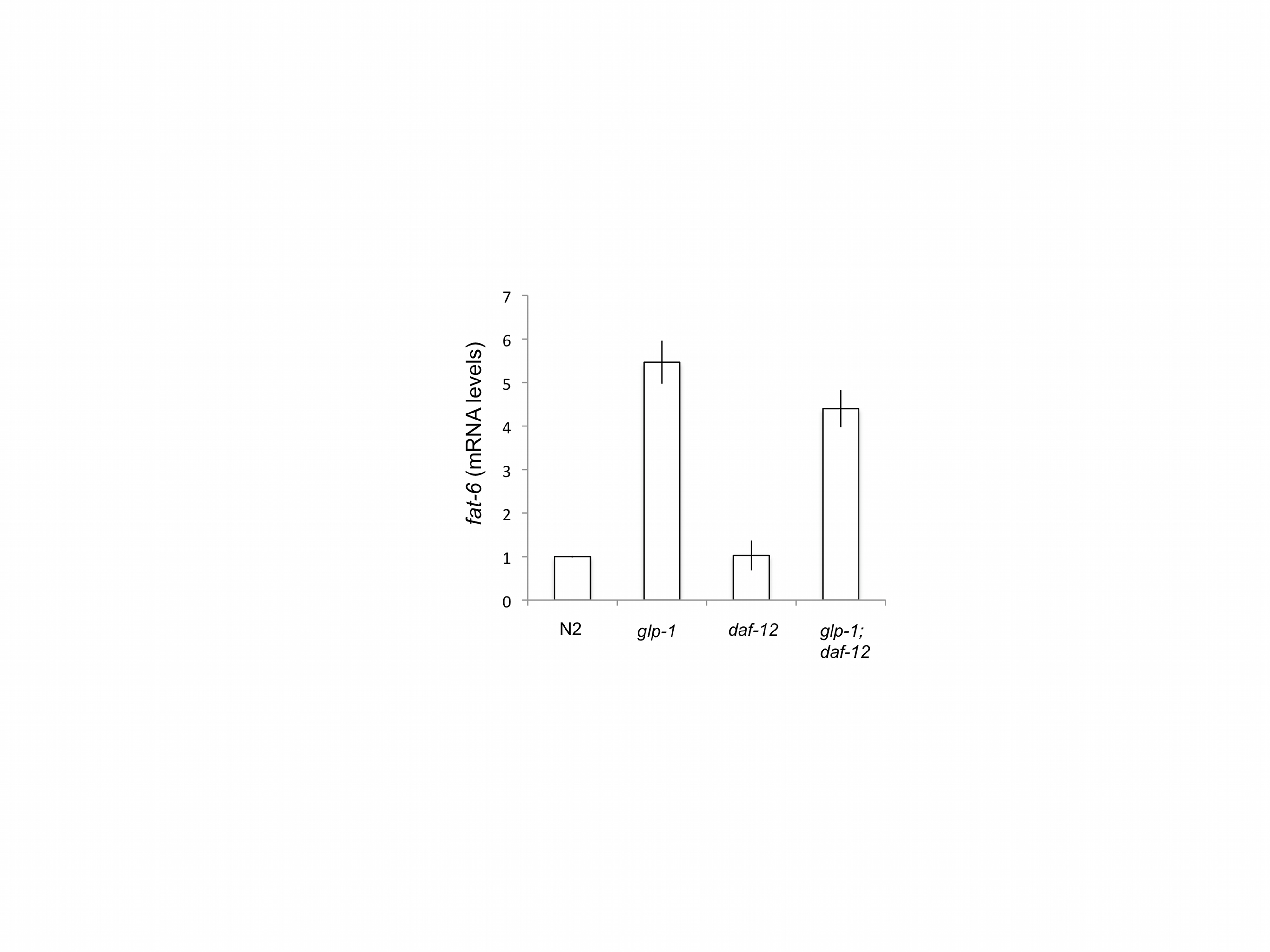

Supplement: Figure S13 — fat-6 expression does not require the presence of daf-12. fat-6 mRNA levels are increased in glp-1(e2141ts) and in glp-1(e2141ts);daf-12(rh61rh411) mutants as measured by qRT-PCR (5.5- and 4.4-fold increase; Wilcoxon rank-sum test p value <0.001 for both strains when compared with N2). When glp-1(e2141ts);daf-12(rh61rh411) mutants are compared to glp-1(e2141ts) mutants, there is an 0.8-fold decrease (Wilcoxon rank-sum test p value is not significant). Error bars are standard deviation. This suggests that fat-6 mRNA levels respond to glp-1(e2141ts) mutants and that part of this response is daf-12 independent. A control experiment shows that fat-6 is not under the control of daf-12 in the wild type background (Wilcoxon rank-sum test p value is non-significant). (5.64 MB TIF) [file pbio.1000599.s013.tif]
