# Supplementary material for: Fatty Acid Desaturation Links Germ Cell Loss to Longevity Through NHR-80/HNF4 in C. elegans
Source: PLoS Biol. 2011 Mar 15;9(3):e1000599. doi: 10.1371/journal.pbio.1000599 (PMC3057950; doi:10.1371/journal.pbio.1000599)
Supplement: Table S1 — Summary of adult lifespan data presented in this work. (0.05 MB PDF) [file pbio.1000599.s014.pdf]

Strain/Treatment	Mean life span ± SEM (days)	% change	Number of animals that died / total	P value vs. control	Figure in text
N2 / HT115	13.5 ± 0.5		88/186		1A
nhr-80(tm1011) / HT115	12 ± 1	-11%	122/151	0.7108	1A
glp-1(e2141ts) / HT115	20 ± 1		124/143		1A
glp-1(e2141ts);nhr-80(tm1011) / HT115	11.5 ± 0.5	-42%	134/156	<0.0001	1A
N2 / HT115	16.5 ± 1.5		93/125		Not shown
nhr-80(tm1011) / HT115	15 ± 1	-11%	114/161	0.8428	Not shown
glp-1(e2141ts) / HT115	20 ± 1		133/150		Not shown
glp-1(e2141ts);nhr-80(tm1011) / HT115	12.5 ± 0.5	-37%	129/150	<0.0001	Not shown
mes-1(bn7) fertile / HT115	19.5 ± 0.5		167/185		1B
mes-1(bn7) fertile / nhr-80 RNAi	13.5 ± 1.5	-30%	149/161	<0.0001	1B
mes-1(bn7) sterile / HT115	22.5 ± 0.5		104/167		1B
mes-1(bn7) sterile / nhr-80 RNAi	16 ± 1	-29%	114/150	<0.0001	1B
mes-1(bn7) fertile / HT115	18 ± 0.5		83/120		Not shown
mes-1(bn7) fertile / nhr-80 RNAi	15.5 ± 1	-14%	92/115	<0.0001	Not shown
mes-1(bn7) sterile / HT115	23 ± 1		74/98		Not shown
mes-1(bn7) sterile / nhr-80 RNAi	15.5 ± 0.5	-33%	98/140	<0.0001	Not shown
daf-2(e1370) / HT115	39.5 ± 0.5		81/127		2A
daf-2(e1370) / nhr-80 RNAi	39 ± 1	-1%	84/128	0.3777	2A
daf-2(e1370) / daf-16 RNAi	18.5 ± 0.5	-53%	97/134	<0.0001	2A
daf-2(e1370) / HT115	41.5 ± 2.5		99/141		Not shown
daf-2(e1370) / nhr-80 RNAi	42.5 ± 1.5	+2%	99/125	0.0255	Not shown
daf-2(e1370) / daf-16 RNAi	19 ± 1	-46%	72/83	<0.0001	Not shown
N2 / HT115	17 ± 1		124/188		2B
N2 bacterial deprivation	20.5 ± 2.5	+20%	157/200	<0.0001	2B
nhr-80(tm1011) / HT115	17 ± 1		116/156		2B
nhr-80(tm1011) / bacterial deprivation	23 ± 1	+35%	115/172	0.0011	2B
N2 / HT115	16.5 ± 1.5		93/125		Not shown
N2 bacterial deprivation	22 ± 1	+35%	19/274	0.0002	Not shown
nhr-80(tm1011) / HT115	15 ± 1		114/161		Not shown
nhr-80(tm1011) bacterial deprivation	19.5 ± 1.5	+30%	33/262	<0.0001	Not shown
N2 / HT115	19.5 ± 0.5		176/183		2C
N2 / cyc-1 RNAi	30 ± 1	+54%	110/146	<0.0001	2C
nhr-80(tm1011) / HT115	15.5 ± 1.5		144/169		2C
nhr-80(tm1011) / cyc-1 RNAi	32 ± 1	+106%	135/165	<0.0001	2C
N2 / HT115	16.5 ± 1.5		93/125		Not shown
N2 / cyc-1 RNAi	20 ± 1	+21%	68/148	<0.0001	Not shown
nhr-80(tm1011) / HT115	15 ± 1		114/160		Not shown
nhr-80(tm1011) / cyc-1 RNAi	19.5 ± 1.5	+30%	102/212	<0.0001	Not shown
N2 non transgenics sibings / HT115	18 ± 1		102/117		4A
N2;LynEx / HT115	20 ± 1	+11%	86/100	0.8799	4A
N2 non transgenics sibings / HT115	18.5 ± 0.5		119/158		Not shown
N2;LynEx / HT115	17.5 ± 0.5	-5%	103/134	0.0670	Not shown
glp-1(e2141ts) non transgenics sibings / HT115	23.5 ± 0.5		124/130		4B
glp-1(e2141ts);LynEx / HT115	37.5 ± 0.5	+60%	70/100	<0.0001	4B
glp-1(e2141ts) non transgenics sibings / HT115	24 ± 1		169/207		Not shown
glp-1(e2141ts);LynEx / HT115	42 ± 1	+75%	67/93	<0.0001	Not shown
glp-1(e2141ts);daf-16(μ86) non transgenics sibings / HT115	10.5 ± 0.5		139/156		5C
glp-1(e2141ts);daf-16(μ86);LynEx / HT115	14.5 ± 0.5	+38%	71/101	<0.0001	5C
glp-1(e2141ts);daf-16(μ86) non transgenics sibings / HT115	10 ± 1		137/160		Not shown
glp-1(e2141ts);daf-16(μ86);LynEx / HT115	13 ± 1	+30%	67/82	<0.0001	Not shown

glp-1(e2141ts);daf-12(rh61rh411) non transgenics sibings / HT115	20.5 ± 1.5		104/125		5D
glp-1(e2141ts);daf-12(rh61rh411);LynEx / HT115	18 ± 1	-12%	71/87	0.0108	5D
glp-1(e2141ts);daf-12(rh61rh411) non transgenics sibings / HT115	16 ± 1		173/187		Not shown
glp-1(e2141ts);daf-12(rh61rh411);LynEx / HT115	20 ± 1	+25%	27/28	0.1974	Not shown
glp-1(e2141ts);daf-12(rh61rh411) non transgenics sibings / HT115	14.5 ± 1.5		101/125		Not shown
glp-1(e2141ts);daf-12(rh61rh411);LynEx / HT115	17 ± 1	+17%	92/125	0.1807	Not shown

glp-1(e2141ts);daf-9(rh50) non transgenics sibings / HT115	10 ± 1		126/150		5E and S5
glp-1(e2141ts);daf-9(rh50);LynEx / HT115	15 ± 1	+50%	47/73	<0.0001	5E
glp-1(e2141ts);daf-9(rh50) non transgenics sibings / HT115	10 ± 1		70/117		Not shown
glp-1(e2141ts);daf-9(rh50);LynEx / HT115	16 ± 1	+60%	63/108	<0.0001	Not shown
glp-1(e2141ts);daf-9(rh50) non transgenics sibings / HT115	10.5 ± 1.5		79/117		Not shown
glp-1(e2141ts);daf-9(rh50);LynEx / HT115	13 ± 1	+24%	30/47	0.0106	Not shown

glp-1(e2141ts) / HT115 / Oleic Acid until Day 1	26 ± 1		148/156		7A
glp-1(e2141ts) / HT115 / Oleic Acid	24 ± 1	-7%	150/160	0.4290	7A
glp-1(e2141ts);fat-6;fat-7 / Oleic Acid until Day 1	14 ± 1	-46%	131/150	<0.0001	7A
glp-1(e2141ts);fat-6;fat-7 / Oleic Acid	22 ± 1	-15%	97/160	<0.0001	7A
glp-1(e2141ts) / HT115 / Oleic Acid until Day 1	21 ± 1		136/174		Not shown
glp-1(e2141ts) / HT115 / Oleic Acid	22.5 ± 0.5	+7%	163/184	0.6833	Not shown
glp-1(e2141ts);fat-6;fat-7 / HT115 / Oleic Acid until Day 1	12 ± 1	-42%	100/135	<0.0001	Not shown
glp-1(e2141ts);fat-6;fat-7 / HT115 / Oleic Acid	17 ± 1	-19%	144/181	0.0038	Not shown

N2 / HT115 / Oleic Acid until Day 1	17 ± 1		91/162		7B
N2 / HT115 / Oleic Acid	15.5 ± 0.5	-8%	92/147	0.0476	7B
fat-6;fat-7 / Oleic Acid until Day 1	17 ± 1	0%	160/182	0.7446	7B
fat-6;fat-7 / Oleic Acid	15.5 ± 0.5	-8%	124/148	0.0048	7B
N2 / HT115 / Oleic Acid until Day 1	18 ± 1		73/154		Not shown
N2 / HT115 / Oleic Acid	20 ± 1	+11%	83/160	0.3542	Not shown
fat-6;fat-7 / HT115 / Oleic Acid until Day 1	16 ± 1	-11%	139/160	<0.0001	Not shown
fat-6;fat-7 /HT115 / Oleic Acid	18 ± 1	0%	135/160	0.2517	Not shown

glp-1(e2141ts) / HT115	26.5 ± 1.5		94/125		Not shown
glp-1(e2141ts) / HT115 / Oleic Acid	24 ± 1	-9%	128/140	0.8034797	Not shown
glp-1(e2141ts);nhr-80 / HT115	11 ± 1		117/130		8A
glp-1(e2141ts);nhr-80 / HT115 / Oleic Acid	13 ± 1	+20%	86/136	0.00172	8A
glp-1(e2141ts) / HT115	20 ± 1		133/150		Not shown
glp-1(e2141ts) / HT115 / Oleic Acid	22 ± 1	+10%	120/151	0.0472	Not shown
glp-1(e2141ts);nhr-80 / HT115	11.5 ± 0.5		129/150		Not shown
glp-1(e2141ts);nhr-80 / HT115 / Oleic Acid	12.5 ± 0.5	+9%	127/129	<0.0001	Not shown

N2 / HT115 / Oleic Acid until Day 1	14 ± 1		88/124		Not shown
N2 / HT115 / Oleic Acid	14 ± 1	0%	108/126	0.9704	Not shown
glp-1(e2141ts) / HT115 / Oleic Acid until Day 1	26.5 ± 0.5		103/125		8B
glp-1(e2141ts) / HT115 / Oleic Acid	23 ± 1	-13%	101/126	0.2547	Not shown
glp-1(e2141ts);fat-6;fat-7 / HT115 / Oleic Acid until Day 1	12 ± 1		104/120		8B
glp-1(e2141ts);fat-6;fat-7 / HT115 / Oleic Acid	18 ± 1	+50%	87/117	<0.0001	8B
glp-1(e2141ts);fat-6;fat-7;LynEx / HT115 / Oleic Acid until Day 1	12 ± 1		64/75		8B
glp-1(e2141ts);fat-6;fat-7;LynEx / HT115 / Oleic Acid	18 ± 1	+50%	63/75	<0.0001	8B
glp-1(e2141ts);fat-6;fat-7 / nhr80 RNAi Oleic Acid until Day 1	7.5 ± 1.5		98/105		8C
glp-1(e2141ts);fat-6;fat-7 / nhr80 RNAi Oleic Acid	7.5 ± 1.5	0%	79/116	0.1312	8C
N2 / HT115 / Oleic Acid until Day 1	16 ± 1		79/120		Not shown
N2 / HT115 / Oleic Acid	17 ± 1	+6%	85/112	0.6550	Not shown
glp-1(e2141ts) / HT115 / Oleic Acid until Day 1	24.5 ± 0.5		90/135		Not shown
glp-1(e2141ts) / HT115 / Oleic Acid	24 ± 1	-2%	98/123	0.9101	Not shown
glp-1(e2141ts);fat-6;fat-7 / HT115 / Oleic Acid until Day 1	13 ± 1		96/122		Not shown
glp-1(e2141ts);fat-6;fat-7 / HT115 / Oleic Acid	18.5 ± 1.5	+42%	91/111	<0.0001	Not shown
glp-1(e2141ts);fat-6;fat-7;LynEx / HT115 / Oleic Acid until Day 1	13 ± 1		85/110		Not shown
glp-1(e2141ts);fat-6;fat-7;LynEx / HT115 / Oleic Acid	18 ± 1	+38%	87/120	<0.0001	Not shown
glp-1(e2141ts);fat-6;fat-7 / nhr80 RNAi Oleic Acid until Day 1	6 ± 1		123/160		Not shown
glp-1(e2141ts);fat-6;fat-7 / nhr80 RNAi Oleic Acid	6 ± 1	0%	120/150	0.2210	Not shown

glp-1(e2141ts) / HT115	26.5 ± 1.5		94/125		S2
glp-1(e2141ts) / nhr-80 RNAi	12.5 ± 0.5	-53%	112/134	<0.0001	S2
glp-1(e2141ts);nhr-80(tm1011) / HT115	11.5 ± 0.5	-57%	117/130	<0.0001	S2
glp-1(e2141ts) / HT115	20 ± 1		133/150		Not shown
glp-1(e2141ts) / nhr-80 RNAi	10.5 ± 1	-47%	122/150	<0.0001	Not shown
glp-1(e2141ts);nhr-80(tm1011) / HT115	11.5 ± 0.5	-42%	129/150	<0.0001	Not shown
glp-1(e2141ts) / HT115	25 ± 1		126/138		S3
glp-1(e2141ts);nhr-80(tm1011) non transgenics sibings / HT115	11 ± 1	-56%	133/144	<0.0001	S3
glp-1(e2141ts);nhr-80(tm1011);LynEx / HT115	25 ± 1	0%	80/107	0.2074	S3
glp-1(e2141ts) / HT115	20 ± 1		76/105		Not shown
glp-1(e2141ts);nhr-80(tm1011) non transgenics sibings / HT115	10 ± 1	-50%	85/102	<0.0001	Not shown
glp-1(e2141ts);nhr-80(tm1011);LynEx / HT115	20 ± 1	0%	34/40	0.9469	Not shown
glp-1(e2141ts);daf-16(m86) / HT115	12 ± 1		89/148		S4
glp-1(e2141ts);daf-16(m86) / nhr-80 RNAi	7 ± 0	-41%	98/135	<0.0001	S4
glp-1(e2141ts);daf-16(m86) / HT115	10.5 ± 1		139/156		Not shown
glp-1(e2141ts);daf-16(m86) / nhr-80 RNAi	6 ± 0	-43%	168/194	<0.0001	Not shown
glp-1(e2141ts);daf-9(rh50) / HT115	10 ± 1		126/150		S5 and 5E
glp-1(e2141ts);daf-9(rh50) / HT115 / Dafachronic Acid	17.5 ± 1.5	+75%	76/125	<0.0001	S5
glp-1(e2141ts) / HT115	20 ± 1	+100%	92/130	<0.0001	S5
glp-1(e2141ts);daf-9(rh50);LynEx / HT115 / Dafachronic Acid	26.5 ± 1.5	+165%	42/93	<0.0001	S5
glp-1(e2141ts);daf-9(rh50) / HT115	10 ± 1		70/117		Not shown
glp-1(e2141ts);daf-9(rh50) / HT115 / Dafachronic Acid	19 ± 1	+90%	67/127	<0.0001	Not shown
glp-1(e2141ts) / HT115	21 ± 0.5	+110%	73/119	<0.0001	Not shown
glp-1(e2141ts);daf-9(rh50);LynEx / HT115 / Dafachronic Acid	27 ± 1	+170%	70/96	<0.0001	Not shown
glp-1(e2141ts) / HT115	27.5 ± 1.5		65/100		S8
glp-1(e2141ts);fat-5(tm420) / HT115	27.5 ± 1.5	0%	107/125	0.9008	S8
glp-1(e2141ts);fat-6(tm331) / HT115	23 ± 1	-16%	83/122	0.1050	S8
glp-1(e2141ts);fat-7(wa36) / HT115	27.5 ± 1.5	0%	104/125	0.8065	S8
glp-1(e2141ts) / HT115	26 ± 1		41/95		Not shown
glp-1(e2141ts);fat-5(tm420) / HT115	23.5 ± 1.5	-9%	105/130	0.1585	Not shown
glp-1(e2141ts);fat-6(tm331) / HT115	21 ± 1	-19%	107/148	0.1309	Not shown
glp-1(e2141ts);fat-7(wa36) / HT115	21 ± 1	-19%	54/137	0.4374	Not shown
glp-1(e2141ts) / HT115	26 ± 1		144/159		S10A
glp-1(e2141ts);fat-5(tm420);fat-6(tm331) / HT115	20 ± 1	-23%	113/143	<0.0001	S10A
glp-1(e2141ts);fat-5(tm420);fat-7(wa36) / HT115	26 ± 1	0%	109/153	0.0005	S10A
glp-1(e2141ts) / HT115	22.5 ± 0.5		113/157		Not shown
glp-1(e2141ts);fat-5(tm420);fat-6(tm331) / HT115	15.5 ± 0.5	-31%	98/164	<0.0001	Not shown
glp-1(e2141ts);fat-5(tm420);fat-7(wa36) / HT115	17 ± 1	-24%	93/163	0.0067	Not shown
N2 / HT115	17 ± 1		91/162		S10B
fat-5(tm420);fat-6(tm331) / HT115	15.5 ± 0.5	-9%	54/165	0.1978	S10B
fat-5(tm420);fat-7(wa36) / HT115	19 ± 1	+12%	74/161	0.0081	S10B
N2 / HT115	20 ± 1		71/158		Not shown
fat-5(tm420);fat-6(tm331) / HT115	18 ± 1	-10%	115/151	<0.0001	Not shown
fat-5(tm420);fat-7(wa36) / HT115	22 ± 1	+10%	112/158	<0.0001	Not shown
N2 / HT115 / Oleic Acid	15.5 ± 0.5		92/147		S10C
fat-5(tm420);fat-6(tm331) / HT115 / Oleic Acid	15.5 ± 0.5	0%	86/160	0.0509	S10C
fat-5(tm420);fat-7(wa36) / HT115 / Oleic Acid	17 ± 1	9%	118/194	0.0227	S10C
N2 / HT115 / Oleic Acid	20 ± 1		83/160		Not shown
fat-5(tm420);fat-6(tm331) / HT115 / Oleic Acid	16 ± 1	-20%	104/141	0.0023	Not shown
fat-5(tm420);fat-7(wa36) / HT115 / Oleic Acid	20 ± 1	0%	65/158	0.0270	Not shown
N2 / HT115	13 ± 1		86/145		S11
N2 / fat-2 RNAi	15.5 ± 1.5	+20%	109/149	0.3285	S11
glp-1(e2141ts) / HT115	29.5 ± 1.5		85/139		S11
glp-1(e2141ts) / fat-2 RNAi	27 ± 1	-8%	52/141	0.5250	S11
glp-1(e2141ts) / HT115	29 ± 2		86/125		Not shown

glp-1(e2141ts) / fat-2 RNAi	23 ± 2	-20%	46/125	0.0389	Not shown
glp-1(e2141ts);daf-16(m86) / HT115	11 ± 1		111/130		S12A
glp-1(e2141ts);daf-16(m86) / HT115 / Oleic Acid	11 ± 1	0%	117/134	0.0871	S12A
glp-1(e2141ts);daf-16(m86) / HT115	10.5 ± 1.5		98/117		Not shown
glp-1(e2141ts);daf-16(m86) / HT115 / Oleic Acid	10.5 ± 1.5	0%	80/122	0.911	Not shown
glp-1(e2141ts);daf-12(rh61rh411) / HT115	20.5 ± 1.5		104/125		S12B
glp-1(e2141ts);daf-12(rh61rh411) / HT115 / Oleic Acid	16 ± 1	-22%	107/124	0.3713	S12B
glp-1(e2141ts);daf-12(rh61rh411) / HT115	17± 1		128/259		Not shown
glp-1(e2141ts);daf-12(rh61rh411) / HT115 / Oleic Acid	17 ± 1	0%	130/151	0.5608	Not shown
glp-1(e2141ts);daf-9(rh50) / HT115	10.5 ± 1.5		103/130		S12C
glp-1(e2141ts);daf-9(rh50) / HT115 / Oleic Acid	10.5 ± 1.5	0%	75/126	0.4388	S12C
glp-1(e2141ts);daf-9(rh50) / HT115	10.5 ± 1.5		79/117		Not shown
glp-1(e2141ts);daf-9(rh50) / HT115 / Oleic Acid	10.5 ± 1.5	0%	81/123	0.0464	Not shown
